# Protocol for a feasibility randomised trial of low-intensity interventions for antenatal depression: ADAGIO trial comparing interpersonal counselling with cognitive behavioural therapy

**DOI:** 10.1136/bmjopen-2019-032649

**Published:** 2019-08-18

**Authors:** Jenny Ingram, Debbie Johnson, Sarah Johnson, Heather A O'Mahen, David Kessler, Hazel Taylor, Roslyn Law, Jeff Round, Jenny Ford, Rebecca Hopley, Joel Glynn, Iryna Culpin, Jonathan Evans

**Affiliations:** 1 Centre for Academic Child Health, Bristol Medical School, University of Bristol, Bristol, UK; 2 University Hospitals Bristol NHS Foundation Trust, Bristol, UK; 3 Department of Psychology, University of Exeter, Exeter, UK; 4 Centre for Academic Mental Health, Bristol Medical School, University of Bristol, Bristol, UK; 5 Anna Freud Centre, London, UK; 6 Institute of Health Economics, Edmonton, Alberta, Canada; 7 Health Economics Bristol, Bristol Medical School, University of Bristol, Bristol, UK

**Keywords:** antenatal depression, cognitive behavioural therapy, interpersonal counselling

## Abstract

**Introduction:**

One in eight women suffer from depression during pregnancy. Currently, low-intensity brief treatment based on cognitive behavioural therapy (CBT) is the only talking treatment widely available in the National Health Service (NHS) for mild and moderate depression. CBT involves identifying and changing unhelpful negative thoughts and behaviours to improve mood. Mothers in our patient advisory groups requested greater treatment choice. Interpersonal counselling (IPC) is a low-intensity version of interpersonal therapy. It may have important advantages during pregnancy over CBT because it targets relationship problems, changes in role and previous losses (eg, miscarriage). We aim to compare CBT and IPC for pregnant women with depression in a feasibility study.

**Methods and analysis:**

A two-arm non-blinded randomised feasibility study of 60 women will be conducted in two UK localities. Women with depression will be identified through midwife clinics and ultrasound scanning appointments and randomised to receive six sessions of IPC or CBT. In every other way, these women will receive usual care. Women thought to have severe depression will be referred for more intensive treatment. After 12 weeks, we will measure women’s mood, well-being, relationship satisfaction and use of healthcare. Women, their partners and staff providing treatments will be interviewed to understand whether IPC is an acceptable approach and whether changes should be introduced before applying to run a larger trial.

Several groups of patients with depression during pregnancy have contributed to our study design. A patient advisory group will meet and advise us during the study.

**Ethics and dissemination:**

Study results will inform the design of a larger multicentre randomised controlled trial (RCT). Our findings will be shared through public engagement events, papers and reports to organisations within the NHS. National Research Ethics Service Committee approved the study protocol.

**Trial registration number:**

ISRCTN11513120.

**Strengths and limitations of this study**

- This study uses a two-centre randomised controlled trial design to determine the feasibility of a definitive trial.
- Two methods of identifying participants will be used: through community midwives at booking and through ultrasound scanning clinics.
- Two talking therapies will be offered to assess the acceptability of these interventions during pregnancy.
- A process evaluation will explore the fidelity of intervention delivery and the experience of women, partners (or a significant other) and therapists.
- A definitive trial would be necessary to evaluate the effectiveness of the intervention.

## Introduction

Antenatal depression is common, with a reported prevalence of 11%^1^ and a point prevalence of up to 44% in certain populations.^2^ Antenatal depression is associated with a range of poor outcomes including continuing depression into the postnatal period, reduced breastfeeding rates,^3^ infant developmental delay^4^ and social/emotional problems, including depression in the offspring during adolescence.^5^ Pregnancy does not appear to be protective against developing depression or relapsing in those with a previous history of depression.^6^

Currently, the Whooley questions are used to identify women who may have antenatal depression.^7^ This screening method has high sensitivity (95%) and modest specificity (65%) for antenatal depression.^8^ However, anecdotal evidence suggests that women are not always aware that they are referred for further help following a positive Whooley screen.^9^

There is limited evidence for the effectiveness of psychological interventions for antenatal depression,^10^ despite a widespread reluctance of mothers to take antidepressants during pregnancy^11^ with 75% of women discontinuing antidepressant medication in the first trimester.^12^ There is also concern among clinicians about prescribing antidepressant medication,^13^ largely due to a lack of evidence regarding their safety,^14^ indicating that psychological interventions are particularly important at this time.^10 15^ One study found that women who discontinue antidepressant medication during pregnancy are five times more likely to experience a relapse in their depression requiring treatment.^6^ The current treatment recommendation for mild to moderate depression, including depression that occurs during pregnancy, is a brief, one-to-one supported self-help approach using the principles of cognitive behavioural therapy (CBT) known as low-intensity CBT.

### Theoretical basis of CBT and limitations for this population

The theoretical basis of CBT is that individuals prone to depression hold dysfunctional beliefs and therefore see the world through a negative filter.^16^ Adversity, such as pregnancy, triggers these underlying beliefs and prone individuals then behave in a way that is consistent with these negative beliefs thus triggering and reinforcing depression. There is no specific relevance of this model to pregnancy and it relies on the skill of the therapist to adapt CBT to individual circumstances. Although there is a growing evidence base for ‘high intensity’ CBT delivered by experienced therapists for perinatal depression, ‘low-intensity’ CBT is briefer and delivered by less experienced practitioners who have limited scope to adapt to the perinatal context and be flexible in its use. The use of high-intensity CBT is limited within current clinical frameworks due to limitations in funding and therapist availability. CBT has few explicit strategies to manage many of the problems that are common for women with antenatal depression, including role transitions and problems in relationships.

A recent review highlighted the need for more personalised therapies to treat perinatal depression, including interpersonal psychotherapy as a plausible treatment option.^17^ Women in our patient advisory group consultations reported that CBT is ‘too clinical’ and ‘inflexible’ with practitioners not appearing to consider the circumstances of pregnancy. This may be one reason why the uptake of CBT treatment for antenatal depression is low. It has been reported that 14% of women with antenatal depression receive psychological treatment and only 5% with antenatal depression achieve remission following treatment.^18^ Women who are pregnant receive lower rates of psychological intervention than those outside the perinatal period (30% vs 50%) despite an increased need for psychological treatment rather than drug treatment at this time.

### Theoretical basis of interpersonal counselling and advantages for treating antenatal depression

Interpersonal counselling (IPC) is a brief treatment which may be more appropriate for addressing the problems that depressed women have during pregnancy and postnatally. It is derived from interpersonal therapy, which holds that interpersonal relationships are a basic human need and attachment to key individuals provides a secure base from which to manage stressful events and conditions.^19^ Problems in interpersonal relationships can trigger symptoms of depression, such as low mood or sleeplessness, and these symptoms further compromise relationships leading to a downward spiral.^20^ This is particularly relevant to pregnancy as conflict in relationships and poor social support are the strongest risk factors for antenatal depression.^21^

IPC offers a more structured version of IPT that promotes understanding of depression through psychoeducation, problem solving and active involvement of the people in the person’s life to provide support and promote recovery. IPC helps individuals develop useful strategies to manage interpersonal conflict and can involve the partner if appropriate. It also focuses on approaches that help manage changes in role, conflict, isolation and loss (ie, miscarriage, stillbirth, termination, previous loss of would be grandparents) and the impact of these on relationships. By directly approaching these issues, IPC addresses what is theoretically central to depression and what service users report are significant worries and concerns for them. The approach is simple and focused and could be provided by practitioners who have limited training or experience.

Currently, there are limited data on the effectiveness of IPC.^14^ It is critical to test its effectiveness because although almost half (43%, n=2 01 591 in 2015–16) of all individuals who receive a talking treatment in the National Health Service (NHS) will receive a brief, ‘low-intensity’ treatment, interpersonal brief treatment approaches are currently not available in the NHS.^22^

IPC has been clearly developed and rigorously manualised as an intervention allowing assessment of fidelity to the model. Although there have been very few studies of the effectiveness of IPC, and none in the UK, one study of depression in primary care in Italy found IPC to be more effective than antidepressants Selective serotonin reuptake inhibitor (SSRIs) particularly for those with less severe depression,^23^ and a small UK pilot study found that IPC is an effective and acceptable treatment for young people with primarily depressive symptoms.^24^ A small feasibility study of IPC for antenatal depression in the USA among low-income mothers indicated high satisfaction with IPC and some improvement in mood.^25^

There are therefore good reasons to hypothesise that IPC may be more acceptable at this time and particularly effective in treating antenatal depression. There is some evidence that interpersonal therapy (IPT), a high-intensity therapy from which IPC is derived is more acceptable than 'high intensity' CBT, with sessions more likely to be attended and that outcomes are better for IPT than 'high intensity' CBT in existing psychological treatment services.^22^ We argue that evaluating this intervention for treating antenatal depression is particularly important because:

1. The model is particularly relevant to pregnanc,y and therefore the advantages of this approach over standard CBT treatment may be greater than at other times.^26^
2. Psychological treatment provision is a priority at this time because of the potential risks of antidepressants during pregnancy and the costs of depression during pregnancy.^27^

Training low-intensity practitioners in IPC has the potential to offer women an acceptable, empirically valid option which may prove to be more effective than CBT and provide the NHS with a treatment option that may be both effective and cost-effective.

### Aims and objectives

Our overall aim is to improve psychological treatment for depression during pregnancy to improve outcomes for the mother, infant and the wider family. We aim to test whether six sessions of IPC are acceptable, effective and cost-effective for treating mild to moderate antenatal depression compared with the most commonly provided existing intervention, low-intensity CBT. Both interventions are one-to-one therapies comprising up to six sessions.

The aim of this first study is to establish the feasibility and acceptability of conducting a full-scale randomised controlled trial (RCT) to compare the effectiveness of these two low-intensity interventions for antenatal depression. Using a pragmatic study design operating within the existing care pathway, we will explore whether a full-scale trial using such a design is feasible. Study objectives are shown in Box 1.

Box 1: **Detailed study objectives**

To determine whether a full trial is feasible by assessing:

- Whether staff who currently deliver low-intensity interventions within routine psychological treatment services, known nationally as Improving Access to Psychological Treatment (IAPT) services, can reliably deliver this newly adapted therapy after brief, additional training.
- Whether it is possible to recruit women from community midwife booking clinics and through screening at the ultrasound scanning clinics.
- Whether it is feasible and acceptable to randomise women following assessment.
- How acceptable IPC is to women, partners and those delivering the intervention relative to low-intensity CBT, assessed through qualitative interviews.
- Whether it is possible to collect sufficient outcome data, including those required to perform an economic evaluation.

## Methods and analysis

### Study design

This feasibility study over 21 months will be carried out in two centres (site A and site B) in preparation for a pragmatic fully powered RCT. Women with mild to moderate depression during pregnancy considered suitable for a low-intensity intervention will be randomised 1:1 either to the usual low-intensity treatment (CBT) or to a novel treatment called IPC.

### Study population, setting and recruitment plan

Women who are aged 18 years or over are eligible for inclusion between 10 and 24 weeks of pregnancy, with an Edinburgh Depression Scale (EPDS) score of 10 or above and mild or moderate depression according to Clinical Interview Schedule Revised (CIS-R),^28^ which gives an International Classification of Diseases 10th Revision diagnosis, whether or not they are taking an antidepressant. Both primiparous and multiparous women will be eligible. Recruitment will continue for 9 months.

We will exclude women with psychotic illness, organic brain disorder, bipolar disorder, personality disorder, alcohol or substance dependency, which will be identified through self-report. Also excluded will be those judged to be at high suicide risk in assessor’s judgement or from response to items on suicide in CIS-R or EPDS, those with severe depression according to CIS-R criteria and those who have had CBT or IPT within the last 6 months. If women miscarry or have a termination during the trial, they will be offered the opportunity to continue with the treatment but will not be included in the rest of the study.

We will compare recruitment through two routes which have been used successfully in two previous trials of antenatal depression and antenatal anxiety.^29 30^

Method 1: Women will be identified at community midwife booking clinics (around 8–10 weeks’ gestation) through the routine screening undertaken for depression using the Whooley questions.^8^ Those who answer ‘yes’ to either question and are considered by the midwife to be appropriate for further assessment for talking therapy, will be asked for their consent to be contacted by the research team about the study.

Method 2: Women will also be identified from ultrasound scan clinics where they will be given study information, eligibility screening questions (EPDS) and a form to indicate their willingness to be contacted by the research team for assessment if screening positive (EPDS 10 or above). This approach will provide a good opportunity to recruit disadvantaged groups, as attendance at scanning clinics does not appear to be socially patterned.^31^

Where needed, interpreters will be used to ensure that those who do not have English as a first language can be offered therapy.

**Figure 1:**
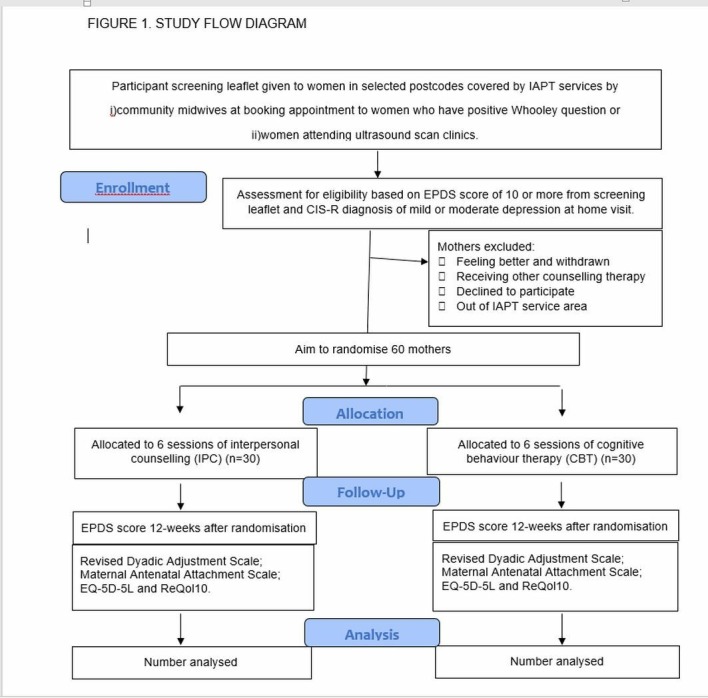
ADAGIO study flow diagram. CBT, cognitivebehavioural therapy; CIS-R, Clinical Interview Schedule Revised; EPDS, Edinburgh Depression Scale; IAPT, Improving Access to Psychological Therapies; IPC, interpersonal counselling.

Figure 1 shows the flow of participants through the trial.

### Assessment and randomisation

A researcher will conduct a telephone assessment with women who consent to be contacted. At a subsequent face-to-face meeting, the researcher will establish eligibility for the trial, obtain written consent and collect baseline data. Partners will also be asked for their consent either in person at the baseline visit, online or by post.

Randomisation will be carried out remotely by Bristol Randomised Trials Collaboration randomisation service. It will be stratified by recruiting centre and minimised by parity (with random block sizes).

### Treatment arms

We have embedded the treatments within existing psychological treatment services available in primary care (Improving Access to Psychological Therapies (IAPT)). For women with mild or moderate depression during pregnancy considered suitable for a low-intensity intervention, we will compare up to six sessions of either low-intensity CBT or IPC.

### Training and supervision for practitioners

To avoid potential selection bias, 12 psychological well-being practitioners will be randomised, 6 to be trained in IPC and 6 to continue delivering low-intensity CBT.

IAPT low-intensity (brief therapy) practitioners who agreed to take part in the study and are randomised to the IPC group will receive 3 days of training from RL (coapplicant; chair of IPT UK; experienced, certified IPT/IPC trainer). Supervisors for the practitioners, who are already trained in IPT (the model from which IPC is derived) will attend the training days. The practitioners will be required to audiorecord cases, which will be assessed by their supervisor to ensure competence and fidelity to the model. Supervision will be provided weekly initially and then fortnightly once practitioners are more familiar with IPC. Those delivering the six sessions of CBT will be given refresher training and have fortnightly case supervision as is usual practice for CBT. Supervisors will rate adherence from a checklist and feedback to trainees. These ratings will be used to assess fidelity.

### Data collection and management

All data collected and analysed during the study will be pseudoanonymised using a unique identifier. A record of trial participants’ names and contact details and assigned trial numbers will be maintained by the trial coordinator and stored separately and securely for administrative purposes. Study data collected by the research team will be recorded on study-specific data collection forms (CRFs). Data will then be entered onto a REDCap database.

### Baseline measurements

Baseline data will be collected at an initial face-to-face assessment with women and will include measures of mood, quality of life, quality of relationship with partner and antenatal attachment. Partners will also be invited to complete a depression rating scale.

### Follow-up measurements

Assessments will be completed either online by participants or over the telephone with a researcher 12 weeks following randomisation. This allows time for women to be allocated to therapy and to complete the sessions. Non-responders will receive two automatic online or telephone reminders 1 week apart, with attempts to collect data by telephone a week later if necessary.

### Measures at baseline and 12 weeks

1. CIS-R a computerised structured psychiatric interview (at baseline only).
2. EPDS^32^ continuous and binary scores from women and their partners. The scale, sometimes known as the Edinburgh Depression Scale, was developed for postnatal depression but is widely used during pregnancy and has been validated outside the postnatal period and for men.^33 34^
3. The Revised Dyadic Adjustment Scale^35^ assesses partner satisfaction.
4. Maternal Antenatal Attachment Scale.^36^
5. Health economic measures outlined below (EQ-5D-5L, ReQol10).
6. At 12 weeks only: the number of sessions attended, number that include the partner, whether step up to more intense psychological intervention is needed, use of medication and use of secondary mental health services.

### Blinding

It will not be possible for assessors or participants to be blind to allocation; however, the statistician will be blind to allocation of participants.

### Outcomes

The primary outcome will be the proportion of eligible women successfully recruited to the point of randomisation.

We will assess numbers and proportions of participants:

- Recruited for assessment (comparing two methods of recruitment).
- Randomised.
- Completing the course of treatment.
- Completing follow-up measures.
- Requiring ‘step up’ to a higher intensity intervention.

We will assess the acceptability of the recruitment method, intervention and study design through a series of in-depth interviews with participants and IAPT practitioners.

The primary outcome for a future trial is likely to be changed in EPDS score, but we will also consider the other secondary outcomes collected in this trial.

### Sample size determination

As this is a feasibility study, the sample size should be sufficient to measure feasibility parameters and data completeness with adequate precision.

There are around 3000 pregnancies per year at site A and 1500 at site B (total 4500) in the relevant IAPT catchment areas, giving a total of approximately 3375 pregnancies in the 9-month recruitment period. Assuming 10% of these have mild–moderate antenatal depression (338) and recruiting through both midwives booking appointments and ultrasound scanning clinics, we aim to include 60 women. This target of 60 subjects from 338 potentially eligible women (17.8%) gives a 95% CI for recruitment between 13.9% and 22.3%.

### Economic evaluation

A full economic evaluation is not possible based on the results of this feasibility study. Within the feasibility study, we assess whether and how necessary data can be collected. We will pilot methods for collecting resource use data in this population and use the results to plan the future main trial. Intervention delivery resource will be recorded.

The main economic outcome measure collected will be the EQ-5D-5L, a generic preference-based measure of health.^37^ Recognising that non-health dimensions of well-being were important to our Patient and Public Involvement (PPI) group, we will also collect data on the ReQoL10 instrument, an alternative preference-based outcome tool that includes domains beyond health and has been developed specifically for use in groups with mental health problems (www.requol.org.uk). Resource use data will be collected online or by telephone as part of the 12-week follow-up. We will ask participants to report resource use during the time enrolled on the study.

### Qualitative study

We will collect qualitative data through interviews to assist in determining the feasibility and acceptability of the intervention and trial design. We will explore the acceptability of the intervention to women receiving IPC and CBT; the feasibility of recruitment and follow-up.

We will conduct semistructured interviews either face to face or on the phone. It is anticipated interviews will last between 30 min and 1 hour. Topic guides will be informed by the research literature, team discussions and input from PPI. We will interview the following groups:

#### i) Women in the treatment arms

These will be conducted at the completion of either IPC (10–12 women) or CBT (5–6 women). Purposive maximum variation sampling will ensure that women from different age groups, socioeconomic status, ethnicity and different levels of engagement with the intervention are selected from both study sites. Interviews will focus on the acceptability and perceived effectiveness of the talking therapy and explore views on the recruitment process; helpful and challenging aspects of the intervention and the appropriateness of the outcome measures being used.

#### ii) Partners (or significant others) of those receiving IPC or CBT

Interviews will be conducted with five or six partners focusing on the acceptability and perceived effectiveness of the intervention and explore ways they feel their partners have benefited from the talking therapy.

#### iii) Participants who drop out

We will seek the views of participants who withdrew or did not attend the intervention to understand their reasons for dropping out and whether continuing participation (and engagement in the intervention) could be supported. These will be short telephone interviews and we would attempt to contact them up to three times.

#### iv) Staff

Interviews (six to eight) with practitioners in the IPC arm, their supervisors and community midwives, will be carried out at the end of the intervention and focus on the acceptability, strengths and weaknesses of the intervention.

### Data analyses

#### Quantitative data analysis

As this is a feasibility trial, no formal statistical testing will be carried out. Instead, the analysis will focus on reporting data that will be used for planning and for assessing the feasibility of the full trial.

A Consolidated Standards of Reporting Trials flow diagram will be produced. Proportions with 95% CIs calculated using the Exact Binomial Method will be produced for:

- Participants consented.
- Participants who are randomised with completed baseline measures.
- Participants randomised to IPC who complete it.
- Participants randomised to low-intensity CBT who complete it.
- Randomised participants lost to follow-up.
- Randomised participants who require ‘step up’ to a higher intensity intervention.
- Randomised participants who have complete outcome data.

Baseline characteristics and demographic characteristics will be tabulated by treatment group (defined by intention to treat) and overall. Means or medians together with appropriate measures of dispersion will be reported for continuous measures and proportions for binary measures. The follow-up outcome data (namely the EPDS, Revised Dyadic Adjustment Scale, Maternal Antenatal Attachment Scale, number of sessions attended, number of sessions partner attended, medication use and secondary health service use) will be reported in the same way. Plots will be used to examine the distribution of continuous outcomes.

#### Qualitative data analysis

All interviews will be audiorecorded, transcribed verbatim and anonymised. Thematic analysis methods^38^ will be used with NVivo to aid data management. Interview transcripts will be read and reread individually, from which an initial coding framework will be developed. Team members will meet to discuss the preliminary coding framework and themes to ensure that the emerging analysis is trustworthy and credible. This framework will be added to and refined, with coded material regrouped as new data from subsequent interviews are gathered.

### Patient and public involvement/patient advisory group

Discussions with women who have perinatal mental health problems and are currently using services, colleagues running voluntary sector perinatal mental health services and feedback from public engagement events have all highlighted the need to improve psychological treatment services available to women and their partners during pregnancy and following childbirth. We asked women attending an antenatal group for those with mental health problems in pregnancy what they thought would have helped them most, and they highlighted the need for a therapy that is more specific to pregnant women. Some reported that current treatment offered (CBT) was ‘too clinical’, and those providing the treatment made little reference to pregnancy or worries about coping with a young baby. We have two collaborators who run voluntary sector organisations providing help to women with mental health problems during the perinatal period. One has been involved in the development of the ideas surrounding this, and the other has been providing advice about women’s experience of local psychological treatment services as well as commenting on the proposal and several aspects of the design. At two public engagement events held in Bristol, we discussed services for families and how these could be improved. At one event for fathers whose partners struggled with their mood and anxiety during pregnancy or following childbirth, they gave a clear message that they felt excluded and even treated with suspicion by services. Fathers welcomed any psychological treatment which might include them and focus on improving the relationship with their partner. Six women, who have been attending either an antenatal group aimed at promoting emotional well-being or postnatal drop-in sessions run by the voluntary sector, have agreed to form a patient advisory group (PAG) meeting three times during the study.

The PAG members will assist in the development of patient facing materials, advise on recruitment issues, inform the development of the topic guide for the qualitative interviews and discuss and help interpret the results including the decision on whether to proceed to a full trial. They will be offered a study-specific induction pack which will include the INVOLVE materials and relevant study information. Training workshops run by People and Research West of England will also be offered to them. PAG members will have their travel expenses and meeting time reimbursed with vouchers. Our findings will be presented in lay terms at a PAG meeting, and they will advise us on routes for dissemination to patient groups.

### Ethics, monitoring and dissemination

This manuscript is based on Protocol V.3.0 dated 14/06/2019. The study received North of Scotland Research Ethics Committee (REC) approval on 29 October 2018 and Health Research Authority approval on 14 November 2018. The trial will be conducted in accordance with the protocol, the principles of the Declaration of Helsinki and International Conference on Harmonisation of technical requirements for registration of pharmaceuticals for human use Good Clinical Practice (ICH GCP). Any amendments of the protocol will be submitted to the REC for approval. On request, the study investigators and their institutions will permit trial-related monitoring and audits by the sponsor and relevant research ethics committee by providing direct access to source data and other documents (ie, patients’ hospital notes). The University of Bristol holds the relevant insurance for this study and is the nominated sponsor for this study.

A trial steering committee (TSC) has been convened to provide overall supervision of the trial and ensure it is in accordance with the principles of good clinical practice and relevant regulations. The TSC agreed the trial protocol and will agree any protocol amendments. The TSC also provides advice to the investigators on all aspects of the trial including aspects of safety and monitoring of serious adverse events. The TSC is chaired by Professor Paul Ramchandani with three independent members who have expertise in clinical psychology and perinatal mental health, midwifery for NHS England and statistics.

### Dissemination

A lay summary of the study is available on the National Institute for Health Research website. Results of this feasibility study will be publicly available through open access publication in a peer-reviewed journals and presented at relevant conferences and research meetings. The PPI groups will contribute to the dissemination plan and assist in the production of the lay summaries.

## Supplementary Material

Reviewer comments

Author's manuscript
