# Supplementary material for: Protocol for a feasibility randomised trial of low-intensity interventions for antenatal depression: ADAGIO trial comparing interpersonal counselling with cognitive behavioural therapy
Source: BMJ Open. 2019 Aug 18;9(8):e032649. doi: 10.1136/bmjopen-2019-032649 (PMC6701625; doi:10.1136/bmjopen-2019-032649)
Supplement: Author's manuscript [file bmjopen-2019-032649.draft_revisions.pdf]

BMJ Open

BMJ Open is committed to open peer review. As part of this commitment we make the peer review history of every article we publish publicly available.

When an article is published we post the peer reviewers' comments and the authors' responses online. We also post the versions of the paper that were used during peer review. These are the versions that the peer review comments apply to.

The versions of the paper that follow are the versions that were submitted during the peer review process. They are not the versions of record or the final published versions. They should not be cited or distributed as the published version of this manuscript.

BMJ Open is an open access journal and the full, final, typeset and author-corrected version of record of the manuscript is available on our site with no access controls, subscription charges or pay-per-view fees (http://bmjopen.bmj.com).

If you have any questions on BMJ Open's open peer review process please email info.bmjopen@bmj.com

BMJ Open

Protocol for a feasibility randomised trial of low intensity interventions for antenatal depression: ADAGIO trial comparing Interpersonal Counselling with Cognitive Behavioural Therapy.

Journal:	*BMJ Open*
Manuscript ID	bmjopen-2019-032649
Article Type:	Protocol
Date Submitted by the Author:	28-Jun-2019
Complete List of Authors:	Ingram, Jenny; University of Bristol, Bristol Medical School, Centre for Academic Child Health<br>Johnson, Debbie; University of Bristol, Bristol Medical School, Centre for Academic Child Health<br>Johnson, Sarah; University Hospitals Bristol NHS Foundation Trust<br>O'Mahen, Heather; University of Exeter<br>Kessler, David; University of Bristol,<br>Taylor, Hazel; University Hospitals Bristol NHS Foundation Trust<br>Law, Roslyn; Anna Freud Centre<br>Round, Jeff; Institute of Health Economics<br>Ford, Jenny<br>Hopley, Rebecca; University of Exeter<br>Glynn, Joel; University of Bristol<br>Culpin, I; University of Bristol, Bristol Medical School<br>Evans, Jonathan; University Of Bristol, Bristol Medical School
Keywords:	MENTAL HEALTH, Depression & mood disorders < PSYCHIATRY, Pregnancy

SCHOLARONE™
Manuscripts

Protocol for a feasibility randomised trial of low intensity interventions for antenatal depression: ADAGIO trial comparing Interpersonal Counselling with Cognitive Behavioural Therapy.

Jenny Ingram[1],

Debbie Johnson[1],

Sarah Johnson[2],

Heather O'Mahen[3],

David Kessler[4],

Hazel Taylor[5],

Roslyn Law[6],

Jeff Round[7],

Jenny Ford[2],

Becky Hopley[3],

Joel Glynn[8],

Iryna Culpin[4],

Jonathan Evans[4]

[1] Centre for Academic Child Health, Bristol Medical School, University of Bristol, UK.

[2] University Hospitals NHS Foundation Trust, Bristol, UK

[3] Department of Psychology, University of Exeter, UK

[4] Centre for Academic Mental Health, Bristol Medical School, University of Bristol, UK.

[5] Research Design Service- South West, University Hospitals NHS Foundation Trust, Bristol, UK

[6] Anna Freud National Centre for Children and Families, London, UK

[7] Institute of Health Economics, Alberta, Canada; and Health Economics Bristol, University of Bristol, UK

[8] Health Economics Bristol, Bristol Medical School, University of Bristol, UK

Corresponding author: Dr Jenny Ingram, Centre for Academic Child Health, University of Bristol, Bristol Medical School, 1-5 Whiteladies Road, Bristol BS8 1NU. 0117 428 3095;

Email: jenny.ingram@bristol.ac.uk.

For peer review only - http://bmjopen.bmj.com/site/about/guidelines.xhtml

ABSTRACT

Introduction. One in eight women suffer from depression during pregnancy. Currently low intensity brief treatment based on cognitive behavioural therapy (CBT) is the only talking treatment widely available in the NHS for mild and moderate depression. CBT involves identifying and changing unhelpful negative thoughts and behaviours to improve mood. Mothers in our patient advisory groups requested greater treatment choice. Interpersonal counselling (IPC)is a low intensity version of interpersonal therapy. It may have important advantages during pregnancy over CBT because it targets relationship problems, changes in role and previous losses (e.g. miscarriage). We aim to compare CBT and IPC for pregnant women with depression in a feasibility study.

Methods and analysis: A two-arm non-blinded randomised feasibility study of 60 women will be conducted in two UK localities. Women with depression will be identified through midwife clinics and ultrasound scanning appointments and randomised to receive six sessions of IPC or CBT. In every other way these women will receive usual care. Women thought to have severe depression will be referred for more intensive treatment. After 12 weeks we will measure women's mood, well-being, relationship satisfaction and use of health care. Women, their partners and staff providing treatments will be interviewed to understand whether IPC is an acceptable approach and whether changes should be introduced before applying to run a larger trial.
Several groups of patients with depression during pregnancy have contributed to our study design. A patient advisory group will meet and advise us during the study.

Ethics and dissemination: Study results will inform the design of a larger multicentre RCT. Our findings will be shared through public engagement events, papers and reports to organisations within the NHS. National Research Ethics Service Committee approved the study protocol.

Trial registration number: ISRCTN 11513120

For peer review only - http://bmjopen.bmj.com/site/about/guidelines.xhtml

Article summary.

Strengths and limitations of the study.

- Psychological treatment provision during pregnancy is important due to the consequences of depression at this time and uncertainty over the use of antidepressants during pregnancy. IPC is particularly relevant to issues that may arise in pregnancy and therefore the advantages of this approach over standard CBT treatment may be greater than at other times.
- This study uses a two-centre randomised controlled trial design to determine the feasibility of a definitive trial.
- A process evaluation will explore the fidelity of intervention delivery and the experience of women, partners (or a significant other) and therapists.
- A definitive trial would be necessary to evaluate the effectiveness of the intervention.

Key words: Antenatal depression; Cognitive behavioural therapy; interpersonal counselling.

For peer review only

For peer review only - http://bmjopen.bmj.com/site/about/guidelines.xhtml

INTRODUCTION.

Antenatal depression is common, with a reported prevalence of 11%[1] and a point prevalence of up to 44% in certain populations.[2] Antenatal depression is associated with a range of poor outcomes including continuing depression into the postnatal period, reduced breastfeeding rates[3], infant developmental delay[4], and social/emotional problems, including depression in the offspring during adolescence.[5] Pregnancy does not appear to be protective against developing depression or relapsing in those with a previous history of depression.[6]

Currently, the Whooley questions are used to identify women who may have antenatal depression.[7] This screening method has high sensitivity (95%) and modest specificity (65%) for antenatal depression.[8] However, anecdotal evidence suggests that women are not always aware that they are referred for further help following a positive Whooley screen.[9]

There is limited evidence for the effectiveness of psychological interventions for antenatal depression[10], despite a widespread reluctance of mothers to take antidepressants during pregnancy[11] with 75% of women discontinuing antidepressant medication in the first trimester.[12] There is also concern amongst clinicians about prescribing antidepressant medication[13], largely due to a lack of evidence regarding their safety[14], indicating that psychological interventions are particularly important at this time.[10,15] One study found that women who discontinue antidepressant medication during pregnancy are five times more likely to experience a relapse in their depression requiring treatment.[6] The current treatment recommendation for mild to moderate depression, including depression that occurs during pregnancy, is a brief, one to one supported self-help approach using the principles of cognitive behavioural therapy (CBT) known as low intensity CBT.

Theoretical basis of CBT and limitations for this population.

The theoretical basis of CBT is that individuals prone to depression hold dysfunctional beliefs and therefore see the world through a negative filter.[16] Adversity, such as pregnancy, triggers these underlying beliefs and prone individuals then behave in a way that is consistent with these negative beliefs thus triggering and reinforcing depression. There is no specific relevance of this model to pregnancy and it relies on the skill of the therapist to adapt CBT to individual circumstances. Although there is a growing evidence base for ‘high intensity’ CBT delivered by experienced therapists for perinatal depression, ‘low intensity’ CBT is briefer and delivered by less experienced practitioners who have limited scope to adapt to the perinatal context and be flexible in its use. The use of high intensity CBT is limited within current clinical frameworks due to limitations in funding and therapist

For peer review only - http://bmjopen.bmj.com/site/about/guidelines.xhtml

availability.CBT has few explicit strategies to manage many of the problems that are common for women with antenatal depression, including role transitions and problems in relationships.

A recent review highlighted the need for more personalized therapies to treat perinatal depression, including interpersonal psychotherapy as a plausible treatment option.[17] Women in our patient advisory group consultations reported that CBT is 'too clinical' and 'inflexible' with practitioners not appearing to consider the circumstances of pregnancy. This may be one reason why the uptake of CBT treatment for antenatal depression is low. It has been reported that 14% of women with antenatal depression receive psychological treatment and only 5% with antenatal depression achieve remission following treatment.[18] Women who are pregnant receive lower rates of psychological intervention than those outside the perinatal period (30% vs 50%) despite an increased need for psychological treatment rather than drug treatment at this time.

Theoretical basis of Interpersonal Counselling (IPC) and advantages for treating antenatal depression.

IPC is a brief treatment which may be more appropriate for addressing the problems that depressed women have during pregnancy and postnatally. It is derived from interpersonal therapy, which holds that interpersonal relationships are a basic human need and attachment to key individuals provides a secure base from which to manage stressful events and conditions.[19] Problems in interpersonal relationships can trigger symptoms of depression, such as low mood or sleeplessness, and these symptoms further compromise relationships leading to a downward spiral.[20] This is particularly relevant to pregnancy as conflict in relationships and poor social support are the strongest risk factors for antenatal depression.[21]

IPC offers a more structured version of IPT that promotes understanding of depression through psychoeducation, problem solving and active involvement of the people in the person's life to provide support and promote recovery. IPC helps individuals develop useful strategies to manage interpersonal conflict and can involve the partner if appropriate. It also focuses on approaches that help manage changes in role, conflict, isolation and loss (i.e., miscarriage, still-birth, termination, previous loss of would be grandparents) and the impact of these on relationships. By directly approaching these issues, IPC addresses what is theoretically central to depression and what service users report are significant worries and concerns for them. The approach is simple and focused and could be provided by practitioners who have limited training or experience.

For peer review only - http://bmjopen.bmj.com/site/about/guidelines.xhtml

Currently there are limited data on the effectiveness of IPC.[14] It is critical to test its effectiveness because although almost half (43%, n=201,591 in 2015-16) of all individuals who receive a talking treatment in the NHS will receive a brief, “low-intensity” treatment, interpersonal brief treatment approaches are currently not available in the NHS.[22]

IPC has been clearly developed and rigorously manualised as an intervention allowing assessment of fidelity to the model. Although there have been very few studies of the effectiveness of IPC, and none in the UK, one study of depression in primary care in Italy found IPC to be more effective than antidepressants (SSRIs) particularly for those with less severe depression.[23] and a small UK pilot study found that IPC is an effective and acceptable treatment for young people with primarily depressive symptoms.[24] A small feasibility study of IPC for antenatal depression in the US amongst low income mothers indicated high satisfaction with IPC and some improvement in mood.[25]

There are therefore good reasons to hypothesise that IPC may be more acceptable at this time and particularly effective in treating antenatal depression. There is some evidence that interpersonal therapy (IPT), a high intensity therapy from which IPC is derived, is more acceptable than 'high intensity' CBT, with sessions more likely to be attended and that outcomes are better for IPT than 'high intensity' CBT in existing psychological treatment services.[22] We argue that evaluating this intervention for treating antenatal depression is particularly important because:
1) the model is particularly relevant to pregnancy and therefore the advantages of this approach over standard CBT treatment may be greater than at other times.[26]
2) psychological treatment provision is a priority at this time because of the potential risks of antidepressants during pregnancy and the costs of depression during pregnancy.[27]

Training low-intensity practitioners in IPC has the potential to offer women an acceptable, empirically valid option which may prove to be more effective than CBT and provide the NHS with a treatment option that may be both effective and cost-effective.

AIMS and OBJECTIVES.

Our overall aim is to improve psychological treatment for depression during pregnancy in order to improve outcomes for the mother, infant and the wider family. We aim to test whether six sessions of IPC are acceptable, effective and cost effective for treating mild to moderate antenatal depression compared to the most commonly provided existing

For peer review only - http://bmjopen.bmj.com/site/about/guidelines.xhtml

intervention, low intensity cognitive behavioural therapy (CBT). Both interventions are one to one therapies comprising up to six sessions.

The aim of this first study is to establish the feasibility and acceptability of conducting a full scale randomised controlled trial to compare the effectiveness of these two low intensity interventions for antenatal depression. Using a pragmatic study design operating within the existing care pathway, we will explore whether a full-scale trial using such a design is feasible. Study objectives are shown in Box 1.

Box 1: Detailed study objectives

To determine whether a full trial is feasible by assessing:

1) Whether staff who currently deliver low intensity interventions within routine psychological treatment services, known nationally as Improving Access to Psychological Treatment (IAPT) services, can reliably deliver this newly adapted therapy after brief, additional training.
2) Whether it is possible to recruit women from community midwife booking clinics and through screening at the ultrasound scanning clinics.
3) Whether it is feasible and acceptable to randomise women following assessment.
4) How acceptable IPC is to women, partners and those delivering the intervention relative to low intensity CBT, assessed through qualitative interviews.
5) Whether it is possible to collect sufficient outcome data, including those required to perform an economic evaluation.

METHODS AND ANALYSIS.

Study design.

This feasibility study over 21 months will be carried out in two centres (site A and site B), in preparation for a pragmatic fully powered RCT. Women with mild to moderate depression during pregnancy considered suitable for a low intensity intervention will be randomised 1:1 either to the usual low intensity treatment (CBT) or to a novel treatment called interpersonal counselling (IPC).

For peer review only - http://bmjopen.bmj.com/site/about/guidelines.xhtml

Study population, setting and recruitment plan

Women who are aged 18 years or over are eligible for inclusion between 10 and 24 weeks of pregnancy, with an Edinburgh Depression Scale (EPDS) score 10 or above and mild or moderate depression according to Clinical Interview Schedule Revised (CIS-R)[28], which gives an ICD-10 diagnosis, whether or not they are taking an antidepressant. Both primiparous and multiparous women will be eligible. Recruitment will continue for 9 months.

We will exclude women with psychotic illness, organic brain disorder, bipolar disorder, personality disorder, alcohol or substance dependency, which will be identified through self-report. Also excluded will be those judged to be at high suicide risk in assessor's judgement or from response to items on suicide in CIS-R or EPDS, those with severe depression according to CIS-R criteria, and those who have had CBT or IPT within the last 6 months. If women miscarry or have a termination during the trial, they will be offered the opportunity to continue with the treatment but will not be included in the rest of the study.

We will compare recruitment through two routes which have been used successfully in two previous trials of antenatal depression and antenatal anxiety:[29, 30]
Method 1: Women will be identified at community midwife booking clinics (around 8-10 weeks' gestation) through the routine screening undertaken for depression using the Whooley questions.[8] Those who answer 'yes' to either question and are considered by the midwife to be appropriate for further assessment for talking therapy, will be asked for their consent to be contacted by the research team about the study.

Method 2: Women will also be identified from ultrasound scan clinics where they will be given study information, eligibility screening questions (EPDS) and a form to indicate their willingness to be contacted by the research team for assessment if screening positive (EPDS 10 or above). This approach will provide a good opportunity to recruit disadvantaged groups, as attendance at scanning clinics does not appear to be socially patterned.[31] Where needed, interpreters will be used to ensure that those who do not have English as a first language can be offered therapy.

Figure 1 shows the flow of participants through the trial.

Assessment and randomisation.

A researcher will conduct a telephone assessment with women who consent to be contacted. At a subsequent face to face meeting the researcher will establish eligibility for

For peer review only - http://bmjopen.bmj.com/site/about/guidelines.xhtml

the trial, obtain written consent and collect baseline data. Partners will also be asked for their consent either in person at the baseline visit, online or by post.

Randomisation will be carried out remotely by Bristol Randomised Trials Collaboration randomisation service. It will be stratified by recruiting centre and minimised by parity (with random block sizes).

Treatment arms

We have embedded the treatments within existing psychological treatment services available in primary care (Improving Access to Psychological Therapies: IAPT). For women with mild or moderate depression during pregnancy considered suitable for a low intensity intervention, we will compare up to six sessions of either low intensity CBT or IPC.

Training and supervision for practitioners

To avoid potential selection bias, 12 psychological wellbeing practitioners will be randomised, six to be trained in IPC and six to continue delivering low intensity CBT.

IAPT low intensity (brief therapy) practitioners who agreed to take part in the study and are randomised to the IPC group will receive three days of training from RL (co-applicant; chair of Interpersonal Therapy (IPT) UK; experienced, certified IPT/IPC trainer). Supervisors for the practitioners, who are already trained in IPT (the model from which IPC is derived) will attend the training days. The practitioners will be required to audio record cases, which will be assessed by their supervisor to ensure competence and fidelity to the model. Supervision will be provided weekly initially, and then fortnightly once practitioners are more familiar with IPC. Those delivering the six sessions of CBT will be given refresher training and have fortnightly case supervision as is usual practice for CBT. Supervisors will rate adherence from a checklist and feedback to trainees. These ratings will be used to assess fidelity.

Data Collection and management

All data collected and analysed during the study will be pseudo-anonymised using a unique identifier. A record of trial participants' names and contact details and assigned trial numbers will be maintained by the trial coordinator and stored separately and securely for administrative purposes. Study data collected by the research team will be recorded on study specific data collection forms (CRFs). Data will then be entered onto a REDCap database.

For peer review only - http://bmjopen.bmj.com/site/about/guidelines.xhtml

Baseline Measurements

Baseline data will be collected at an initial face-to-face assessment with women and will include measures of mood, quality of life, quality of relationship with partner and antenatal attachment. Partners will also be invited to complete a depression rating scale.

Follow-up Measurements

Assessments will be completed either online by participants or over the telephone with a researcher 12 weeks following randomisation. This allows time for women to be allocated to therapy and to complete the sessions. Non-responders will receive two automatic online or telephone reminders one week apart, with attempts to collect data by telephone a week later if necessary.

Measures at baseline and 12-weeks

1) CIS-R a computerised structured psychiatric interview (at baseline only)
2) Edinburgh Postnatal Depression Scale (EPDS)[32] continuous and binary scores from women and their partners. The scale, sometimes known as the Edinburgh Depression Scale, was developed for postnatal depression but is widely used during pregnancy and has been validated outside the postnatal period and for men.[33, 34]
3) The Revised Dyadic Adjustment Scale[35] assesses partner satisfaction.
4) Maternal Antenatal Attachment Scale.[36]
5) Health economic measures outlined below (EQ-5D-5L, ReQol10).
6) At 12-weeks only: The number of sessions attended, number that include the partner, whether step up to more intense psychological intervention is needed, use of medication, and use of secondary mental health services.

Blinding

It will not be possible for assessors or participants to be blind to allocation however the statistician will be blind to allocation of participants.

Outcomes

The primary outcome will be the proportion of eligible women successfully recruited to the point of randomisation.

We will assess numbers and proportions of participants:

- Recruited for assessment (comparing two methods of recruitment),
- Randomised
- Completing the course of treatment

For peer review only - http://bmjopen.bmj.com/site/about/guidelines.xhtml

• Completing follow up measures
• Requiring 'step up' to a higher intensity intervention.

We will assess the acceptability of the recruitment method, intervention and study design through a series of in-depth interviews with participants and IAPT practitioners.

The primary outcome for a future trial is likely to be change in EPDS score but we will also consider the other secondary outcomes collected in this trial.

Sample size determination.

As this is a feasibility study, the sample size should be sufficient to measure feasibility parameters and data completeness with adequate precision.

There are around 3000 pregnancies per year at site A and 1500 at site B (total 4500) in the relevant IAPT catchment areas, giving a total of approximately 3375 pregnancies in the nine-month recruitment period. Assuming 10% of these have mild-moderate antenatal depression (338) and recruiting through both midwives booking appointments and ultrasound scanning clinics we aim to include 60 women. This target of 60 subjects from 338 potentially eligible women (17.8%) gives a 95% confidence interval for recruitment between 13.9% and 22.3%

Economic evaluation

A full economic evaluation is not possible based on the results of this feasibility study. Within the feasibility study we assess whether and how necessary data can be collected. We will pilot methods for collecting resource use data in this population and use the results to plan the future main trial. Intervention delivery resource will be recorded.

The main economic outcome measure collected will be the EQ-5D-5L, a generic preference-based measure of health.[37] Recognising that non-health dimensions of wellbeing were important to our PPI group, we will also collect data on the ReQoL10 instrument, an alternative preference-based outcome tool that includes domains beyond health and has been developed specifically for use in groups with mental health problems (www.requol.org.uk). Resource use data will be collected online or by telephone as part of the 12-week follow-up. We will ask participants to report resource use during the time enrolled on the study.

For peer review only - http://bmjopen.bmj.com/site/about/guidelines.xhtml

Qualitative study.

We will collect qualitative data through interviews to assist in determining the feasibility and acceptability of the intervention and trial design. We will explore the acceptability of the intervention to women receiving IPC and CBT; the feasibility of recruitment and follow up. We will conduct semi-structured interviews either face to face or on the phone. It is anticipated interviews will last between 30 minutes and 1 hour. Topic guides will be informed by the research literature, team discussions and input from PPI. We will interview the following groups:

A) *Women in the treatment arms*.

These will be conducted at the completion of either IPC (10-12 women) or CBT (5-6 women). Purposive maximum variation sampling will ensure that women from different age groups, socioeconomic status, ethnicity and different levels of engagement with the intervention are selected from both study sites. Interviews will focus on the acceptability and perceived effectiveness of the talking therapy and explore views on the recruitment process; helpful and challenging aspects of the intervention; and the appropriateness of the outcome measures being used.

B) *Partners (or significant others) of those receiving IPC or CBT.*

Interviews will be conducted with 5 or 6 partners focusing on the acceptability and perceived effectiveness of the intervention and explore ways they feel their partners have benefited from the talking therapy.

C) *Participants who drop out.*

We will seek the views of participants who withdrew or did not attend the intervention to understand their reasons for dropping out and whether continuing participation (and engagement in the intervention) could be supported. These will be short telephone interviews and we would attempt to contact them up to three times.

D) *Staff interviews.*

Interviews (6-8) with practitioners in the IPC arm, their supervisors and community midwives, will be carried out at the end of the intervention and focus on the acceptability, strengths and weaknesses of the intervention.

For peer review only - http://bmjopen.bmj.com/site/about/guidelines.xhtml

Data Analyses

Quantitative Data analysis

As this is a feasibility trial no formal statistical testing will be carried out. Instead the analysis will focus on reporting data that will be used for planning and for assessing the feasibility of the full trial.

A CONSORT flow diagram will be produced. Proportions with 95% confidence intervals calculated using the Exact Binomial Method will be produced for:

- Participants consented.
- Participants who are randomised with completed baseline measures.
- Participants randomised to IPC who complete it.
- Participants randomised to low intensity CBT who complete it.
- Randomised participants lost to follow-up.
- Randomised participants who require 'step up' to a higher intensity intervention.
- Randomised participants who have complete outcome data.

Baseline characteristics and demographic characteristics will be tabulated by treatment group (defined by intention to treat) and overall. Means or medians together with appropriate measures of dispersion will be reported for continuous measures and proportions for binary measures. The follow-up outcome data (namely the EPDS, Revised Dyadic Adjustment Scale, Maternal Antenatal Attachment Scale, number of sessions attended, number of sessions partner attended, medication use and secondary health service use) will be reported in the same way. Plots will be used to examine the distribution of continuous outcomes.

Qualitative data analysis

All interviews will be audio-recorded, transcribed verbatim and anonymised. Thematic analysis methods[38] will be used with NVivo to aid data management. Interview transcripts will be read and re-read individually, from which an initial coding framework will be developed. Team members will meet to discuss the preliminary coding framework and themes to ensure that the emerging analysis is trustworthy and credible. This framework will be added to and refined, with coded material regrouped as new data from subsequent interviews are gathered.

For peer review only - http://bmjopen.bmj.com/site/about/guidelines.xhtml

Patient and public involvement / Patient Advisory Group (PAG)

Discussions with women who have perinatal mental health problems and are currently using services, colleagues running voluntary sector perinatal mental health services and feedback from public engagement events, have all highlighted the need to improve psychological treatment services available to women and their partners during pregnancy and following childbirth. We asked women attending an antenatal group for those with mental health problems in pregnancy what they thought would have helped them most and they highlighted the need for a therapy that is more specific to pregnant women. Some reported that current treatment offered (CBT) was 'too clinical' and those providing the treatment made little reference to pregnancy or worries about coping with a young baby. We have two collaborators who run voluntary sector organisations providing help to women with mental health problems during the perinatal period. One has been involved in the development of the ideas surrounding this and the other has been providing advice about women's experience of local psychological treatment services, as well as commenting on the proposal and several aspects of the design. At two public engagement events held in Bristol we discussed services for families and how these could be improved. At one event for fathers whose partners struggled with their mood and anxiety during pregnancy or following child birth they gave a clear message that they felt excluded and even treated with suspicion by services. Fathers welcomed any psychological treatment which might include them and focus on improving the relationship with their partner. Six women who have been attending either an antenatal group aimed at promoting emotional wellbeing or postnatal drop-in sessions run by the voluntary sector, have agreed to form a Patient Advisory Group (PAG) meeting three times during the study.

The PAG members will assist in the development of patient facing materials, advise on recruitment issues, inform the development of the topic guide for the qualitative interviews and discuss and help interpret the results including the decision on whether to proceed to a full trial. They will be offered a study specific induction pack which will include the INVOLVE materials and relevant study information. Training workshops run by People and Research West of England will also be offered to them. PAG members will have their travel expenses and meeting time reimbursed with vouchers. Our findings will be presented in lay terms at a PAG meeting and they will advise us on routes for dissemination to patient groups.

For peer review only - http://bmjopen.bmj.com/site/about/guidelines.xhtml

ETHICS, MONITORING AND DISSEMINATION

This manuscript is based on Protocol V.3.0 dated 21/06/2019. The study received North of Scotland Research Ethics Committee (REC) approval on October 29th 2018 and Health Research Authority approval on November 14th 2018. The trial will be conducted in accordance with the protocol, the principles of the Declaration of Helsinki and ICH GCP. Any amendments of the protocol will be submitted to the REC for approval. On request, the study investigators and their institutions will permit trial-related monitoring and audits by the Sponsor and relevant Research Ethics Committee by providing direct access to source data and other documents (i.e. patients' hospital notes).

A Trial Steering Committee (TSC) has been convened to provide overall supervision of the trial and ensure it is in accordance with the principles of good clinical practice and relevant regulations. The TSC agreed the trial protocol and will agree any protocol amendments. The TSC also provide advice to the investigators on all aspects of the trial including aspects of safety and monitoring of serious adverse events. The TSC is chaired by Prof Paul Ramchandani with three independent members who have expertise in clinical psychology and perinatal mental health, midwifery for NHS England, and statistics.

Dissemination.

A lay summary of the study is available on the NIHR website. Results of this feasibility study will be publicly available through open access publication in a peer-reviewed journals and presented at relevant conferences and research meetings. The PPI groups will contribute to the dissemination plan and assist in the production of the lay summaries.

Word count 3957

Acknowledgements The authors would like to thank the lay representatives who contribute to the trial management and steering groups and the women who contributed to the intervention design.

Author Contributions: JE is the chief investigator and JI drafted the paper with critical input from all authors. All authors contributed to study design and development. JE, JI, HO'M, DK, HT, JR, RL, IC and JF contributed to the inception of the study. All authors read, commented on and approved the final manuscript.

For peer review only - http://bmjopen.bmj.com/site/about/guidelines.xhtml

Funding: This work is supported by the National Institute for Health Research (NIHR) Research for Patient Benefit research programme grant number PB-PG-1216-20027.

Disclaimer: The views expressed are those of the authors and not necessarily those of the NHS, the NIHR or the Department of Health.

Competing interests: None

Provenance and peer review: Not commissioned; externally peer reviewed.

Data sharing statement: The results of this feasibility study will inform the planning of a definitive randomised controlled trial, including whether it can be conducted, by assessing rates of recruitment, retention and data collection.

Trial status: Ongoing data collection.

Trial sponsor: University of Bristol. Bristol BS1 5DD: research-governance@bristol.ac.uk

REFERENCES.

1. Howard, L.M., Ryan, E.G., Trevillion, K., Anderson, F., Bick, D., Bye, A. et al. (2018). Accuracy of the Whooley questions and the Edinburgh Postnatal Depression Scale in identifying depression and other mental disorders in early pregnancy. British Journal of Psychiatry, 212: 50-56.

2. Gavin, N. I., Gaynes, B. N., Lohr, K. N., Meltzer-Brody, S., Gartlehner, G., & Swinson, T. (2005). Perinatal depression: a systematic review of prevalence and incidence. *Obstetrics & Gynecology*, *106*(5, Part 1), 1071-1083.

3. Grigoriadis, S., Vonderporten, E.H., Mamisashvili, L., Tomlinson, G., Dennis, C.L., Koren, G. et al. (2013). The impact of maternal depression during pregnancy on perinatal outcomes: A systematic review and meta-analysis. Journal of Clinical Psychiatry, 74(4): 321

4. Pedersen, L.H., Henriksen, T.B. and Olsen, J. (2010) Fetal exposure to antidepressants and normal milestone development at 6 and 19 months of age. Pediatrics, 125(3): 600-608

5. Madigan, S., Oatley, H., Racine, N., Fearon, R.M.P., Schumacher, L., Akbari, E. et al. (2018). A meta-analysis of maternal prenatal depression and anxiety on child socioemotional development. Journal of the American Academy of Child and Adolescent Psychiatry, 57(9): 645

6. Cohen, L.S., Altshuler, L.L., Harlow, B.L., Nonacs, R., Newport, D.J., Viguera, A.C. et al. (2006). Relapse of major depression during pregnancy in women who maintain or

For peer review only - http://bmjopen.bmj.com/site/about/guidelines.xhtml

For peer review only

discontinue antidepressant treatment. Journal of the American Medical Association, 295(5): 499-507.

7. NICE Clinical guideline 192 (2014) Antenatal and postnatal mental health: clinical management and service guidance.

8. Bosanquet, K., Bailey, D., Gilbody, S., Harden, M., Manea, H., Nutbrown, S. and McMillan, D. (2015). Diagnostic accuracy of the Whooley questions for the identification of depression: A diagnostic meta-analysis. *British Medical Journal Open.* 5(12)

9. Darwin, Z., McGowan, L. and Edozien, L.C. (2015). Antenatal mental health referrals: Review of local clinical practice and pregnant women's experiences in England. Midwifery, 31(3): 17-22

10. Dennis, C. L., Ross, L. E., & Grigoriadis, S. (2007). Psychosocial and psychological interventions for treating antenatal depression. Cochrane Database of Systematic Reviews 2007, Issue 3. Art. No.: CD006309. DOI: 10.1002/14651858.CD006309.pub2.

11. Arch, J.J. (2014). Cognitive behavioral therapy and pharmacotherapy for anxiety: Treatment preferences and credibility among pregnant and non-pregnant women. *Behaviour Research and Therapy* 52:53-60

12. Hayes, R.M., Wu, P.S., Shelton, R.C., Cooper, W.O., Dupont, W.D., Mitchel, E. and Hartert, T.V. (2012) Maternal antidepressant use and adverse outcomes: A cohort study of 228876 pregnancies. American Journal of Obstetrics and Gynecology, 207(1)

13. Bilszta, J.L.C., Tsuchiya, S., Han, K., Buist, A.E., Einarson, A. (2011). Primary care physicians' attitudes and practices regarding antidepressant use during pregnancy: A survey of two countries. *Archives of Womens Mental Health.* 14(1): 71-75

14. Chisolm, M.S. and Payne, J.L. (2016) Management of psychotropic drugs during pregnancy. British Medical Journal, 352:h5918

15. Sockol, L. E., Epperson, C. N., & Barber, J. P. (2011). A meta-analysis of treatments for perinatal depression. Clinical Psychology Review, 31(5), 839-849.

16. Beck, A.T. (1967) Depression: Clinical, Experimental, and Theoretical Aspects. New York: Harper & Row.

17. Johansen, S.L., Robakis, T.K., Williams, K.E. and Rasgon, N.L. (2019) Management of perinatal depression with non-drug interventions. British Medical Journal, 364:l322.

18. Cox, E.Q., Sowa, N.A., Meltzer-Brody, S.E. and Gaynes, B.N. (2016) The perinatal depression treatment cascade: Baby steps toward improving outcomes. Journal of Clinical Psychiatry, 77(9):1189-1200.

19. Ravitz, P., Watson, P., Lawson, A., Contatino, M.J., Bernecker, S., Park, J. and Swartz, H.A. (2019) Interpersonal psychotherapy: A scoping review and historical perspective (1974-2017). Harvard Review of Psychiatry. Accessed online via PMID 30883446.

20. Lipsitz, J. D., & Markowitz, J. C. (2013). Mechanisms of change in interpersonal therapy (IPT). Clinical psychology review, 33(8), 1134-1147.

For peer review only - http://bmjopen.bmj.com/site/about/guidelines.xhtml

21. Lancaster, C. A., Gold, K. J., Flynn, H. A., Yoo, H., Marcus, S. M., & Davis, M. M. (2010). Risk factors for depressive symptoms during pregnancy: a systematic review. American Journal of Obstetrics and Gynecology, 202(1), 5-14.

22. NHS Digital 2016: Psychological Therapies: Annual Report on the use of IAPT services, England, 2015-16

23. Menchetti, M., Rucci, P., Bortolotti, B., Bombi, A., Scocco, P., Kraemer, H. C.,Berardi D, DEPICS group. (2013). Moderators of remission with interpersonal counselling or drug treatment in primary care patients with depression: randomised controlled trial. The British Journal of Psychiatry. 204(2): 144-150.

24. Wilkinson PO, Cestaro V, Pinchen I. Pilot mixed-methods evaluation of interpersonal counselling for young people with depressive symptoms in non-specialist services *Evidence-Based Mental Health* 2018;**21:**134-138.

25. Lenze, Shannon N., and Mary Anne Potts. (2017) Brief Interpersonal Psychotherapy for depression during pregnancy in a low-income population: A randomized controlled trial Journal of Affective Disorders 210: 151-157.

26. Stuart, S. (2012). Interpersonal psychotherapy for postpartum depression. Clinical Psychology & Psychotherapy, 19(2), 134-140.

27. Bauer, A., Parsonage, M., Knapp, M., Lemmi, V., & Adelaja, B. (2014). Costs of perinatal mental health problems. London School of Economics and Political Science.

28. Lewis G, Pelosi AJ, Araya R, Dunn G: (1992) Measuring psychiatric-disorder in the community – a standardized assessment for Use by Lay interviewers. Psychol Med. 22 (2): 465-486.

29. Burns, A., O'Mahen, H., Baxter, H., Bennert, K., Wiles, N., Ramchandani, P., ... & Evans, J. (2013). A pilot randomised controlled trial of cognitive behavioural therapy for antenatal depression.BMC Psychiatry, 13(1), 33.

30. Wilkinson, E. L., O'Mahen, H. A., Fearon, P., Halligan, S., King, D. X., Greenfield, G., ... & Ramchandani, P. G. (2016). Adapting and testing a brief intervention to reduce maternal anxiety during pregnancy (ACORN): study protocol for a randomised controlled trial. Trials, 17(1), 156.

31. Redshaw, M., & Henderson, J. (2013). Fathers' engagement in pregnancy and childbirth: evidence from a national survey. BMC Pregnancy and Childbirth, 13(1), 70.

32. Cox, J. L., Holden, J. M., & Sagovsky, R. (1987). Detection of postnatal depression. Development of the 10-item Edinburgh Postnatal Depression Scale. The British Journal of Psychiatry, 150(6), 782-786.

33. Hewitt C, Gilbody S, Brealey S, et al. (2009) Methods to identify postnatal depression in primary care: an integrated evidence synthesis and value of information analysis. Health Technol Assess.13(36):1-145, 147-230.

34. Edmondson OJ, Psychogiou L, Vlachos H, Netsi E, Ramchandani PG. (2010) Depression in fathers in the postnatal period: assessment of the Edinburgh Postnatal Depression Scale as a screening measure. J Affect Disord.125(1-3):365-368.

For peer review only - http://bmjopen.bmj.com/site/about/guidelines.xhtml

35. Busby DM, Christensen C, Crane DR, Larson JH. (1995). A revision of the dyadic adjustment scale for use with distressed and nondistressed couples: construct hierarchy and multidimensional scales. Journal of Marital and Family Therapy; 21(3):289-308.

36. Condon JT. (1993) The assessment of antenatal emotional attachment: development of a questionnaire instrument. Br J Med Psychol; 66 (Pt 2): 167-183

37. Herdman, M., Gudex, C., Lloyd, A., Janssen, M., Kind, P., Parkin, D., Bonsel, G., Badia, X. (2011) Development and preliminary testing of the new five-level version of EQ-5D (EQ-5D-5L) *Quality of Life Research,* 20(10), pp1727-1736.

38. Braun, V., & Clarke, V. (2006), Using thematic analysis in psychology. *Qualitative Research in Psychology*, *3*, 77-101.

For peer review only

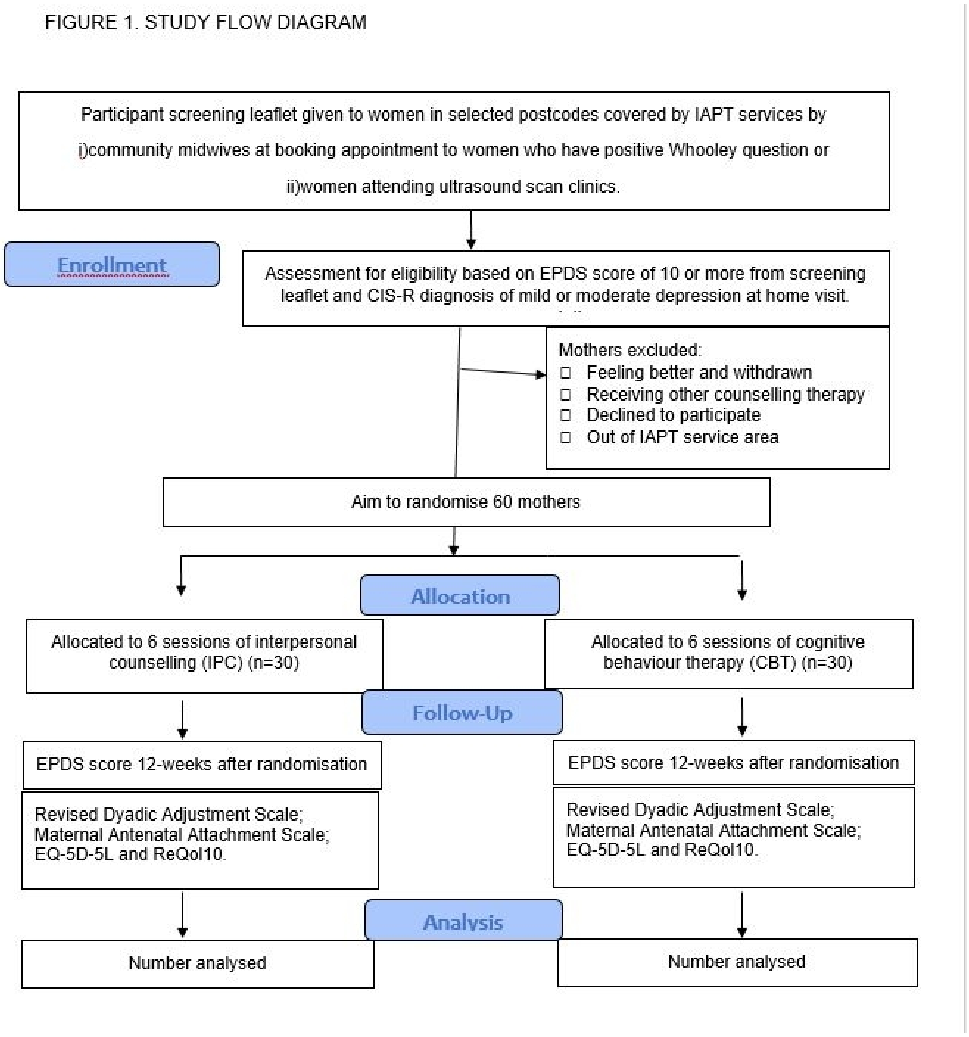

Figure 1. Study flow diagram

239x246mm (72 x 72 DPI)

STANDARD PROTOCOL ITEMS: RECOMMENDATIONS FOR INTERVENTIONAL TRIALS

SPIRIT 2013 Checklist: Recommended items to address in a clinical trial protocol and related documents*

Section/item	Item No	Description	Addressed on page number
**Administrative information**			
Title	1	Descriptive title identifying the study design, population, interventions, and, if applicable, trial acronym	___1_________
Trial registration	2a	Trial identifier and registry name. If not yet registered, name of intended registry	__2___________
	2b	All items from the World Health Organization Trial Registration Data Set	_____________
Protocol version	3	Date and version identifier	___15__________
Funding	4	Sources and types of financial, material, and other support	__16___________
Roles and responsibilities	5a	Names, affiliations, and roles of protocol contributors	___15__________
	5b	Name and contact information for the trial sponsor	___16__________
	5c	Role of study sponsor and funders, if any, in study design; collection, management, analysis, and interpretation of data; writing of the report; and the decision to submit the report for publication, including whether they will have ultimate authority over any of these activities	______
	5d	Composition, roles, and responsibilities of the coordinating centre, steering committee, endpoint adjudication committee, data management team, and other individuals or groups overseeing the trial, if applicable (see Item 21a for data monitoring committee)	__15________

For peer review only

Introduction

Background and rationale	6a	Description of research question and justification for undertaking the trial, including summary of relevant studies (published and unpublished) examining benefits and harms for each intervention	_4-6_________
	6b	Explanation for choice of comparators	__6___________
Objectives	7	Specific objectives or hypotheses	6-7_________
Trial design	8	Description of trial design including type of trial (eg, parallel group, crossover, factorial, single group), allocation ratio, and framework (eg, superiority, equivalence, noninferiority, exploratory)	_7 (in Methods)_

Methods: Participants, interventions, and outcomes

Study setting	9	Description of study settings (eg, community clinic, academic hospital) and list of countries where data will be collected. Reference to where list of study sites can be obtained	_7________
Eligibility criteria	10	Inclusion and exclusion criteria for participants. If applicable, eligibility criteria for study centres and individuals who will perform the interventions (eg, surgeons, psychotherapists)	_8
Interventions	11a	Interventions for each group with sufficient detail to allow replication, including how and when they will be administered	7, 9__
	11b	Criteria for discontinuing or modifying allocated interventions for a given trial participant (eg, drug dose change in response to harms, participant request, or improving/worsening disease)	_8___________
	11c	Strategies to improve adherence to intervention protocols, and any procedures for monitoring adherence (eg, drug tablet return, laboratory tests)	_9_______
	11d	Relevant concomitant care and interventions that are permitted or prohibited during the trial	_n/a___________ _
Outcomes	12	Primary, secondary, and other outcomes, including the specific measurement variable (eg, systolic blood pressure), analysis metric (eg, change from baseline, final value, time to event), method of aggregation (eg, median, proportion), and time point for each outcome. Explanation of the clinical relevance of chosen efficacy and harm outcomes is strongly recommended	__10-11_______

For peer review only - http://bmjopen.bmj.com/site/about/guidelines.xhtml

Participant timeline	13	Time schedule of enrolment, interventions (including any run-ins and washouts), assessments, and visits for participants. A schematic diagram is highly recommended (see Figure)	_8-11_________
Sample size	14	Estimated number of participants needed to achieve study objectives and how it was determined, including clinical and statistical assumptions supporting any sample size calculations	_11__________
Recruitment	15	Strategies for achieving adequate participant enrolment to reach target sample size	__8___________

Methods: Assignment of interventions (for controlled trials)

Allocation:

Sequence generation	16a	Method of generating the allocation sequence (eg, computer-generated random numbers), and list of any factors for stratification. To reduce predictability of a random sequence, details of any planned restriction (eg, blocking) should be provided in a separate document that is unavailable to those who enrol participants or assign interventions	__9___________
Allocation concealment mechanism	16b	Mechanism of implementing the allocation sequence (eg, central telephone; sequentially numbered, opaque, sealed envelopes), describing any steps to conceal the sequence until interventions are assigned	__9___________
Implementation	16c	Who will generate the allocation sequence, who will enrol participants, and who will assign participants to interventions	___8-9_______
Blinding (masking)	17a	Who will be blinded after assignment to interventions (eg, trial participants, care providers, outcome assessors, data analysts), and how	_10__
	17b	If blinded, circumstances under which unblinding is permissible, and procedure for revealing a participant's allocated intervention during the trial	___na__________

Methods: Data collection, management, and analysis

Data collection methods	18a	Plans for assessment and collection of outcome, baseline, and other trial data, including any related processes to promote data quality (eg, duplicate measurements, training of assessors) and a description of study instruments (eg, questionnaires, laboratory tests) along with their reliability and validity, if known. Reference to where data collection forms can be found, if not in the protocol	_10-11__

	18b	Plans to promote participant retention and complete follow-up, including list of any outcome data to be collected for participants who discontinue or deviate from intervention protocols	__10___________
Data management	19	Plans for data entry, coding, security, and storage, including any related processes to promote data quality (eg, double data entry; range checks for data values). Reference to where details of data management procedures can be found, if not in the protocol	___9__________
Statistical methods	20a	Statistical methods for analysing primary and secondary outcomes. Reference to where other details of the statistical analysis plan can be found, if not in the protocol	_13_______
	20b	Methods for any additional analyses (eg, subgroup and adjusted analyses)	_na__________
	20c	Definition of analysis population relating to protocol non-adherence (eg, as randomised analysis), and any statistical methods to handle missing data (eg, multiple imputation)	_____________
**Methods: Monitoring**			
Data monitoring	21a	Composition of data monitoring committee (DMC); summary of its role and reporting structure; statement of whether it is independent from the sponsor and competing interests; and reference to where further details about its charter can be found, if not in the protocol. Alternatively, an explanation of why a DMC is not needed	_15______
	21b	Description of any interim analyses and stopping guidelines, including who will have access to these interim results and make the final decision to terminate the trial	_na____________
Harms	22	Plans for collecting, assessing, reporting, and managing solicited and spontaneously reported adverse events and other unintended effects of trial interventions or trial conduct	____________
Auditing	23	Frequency and procedures for auditing trial conduct, if any, and whether the process will be independent from investigators and the sponsor	_____________
**Ethics and dissemination**			
Research ethics approval	24	Plans for seeking research ethics committee/institutional review board (REC/IRB) approval	_15____________

Protocol amendments	25	Plans for communicating important protocol modifications (eg, changes to eligibility criteria, outcomes, analyses) to relevant parties (eg, investigators, REC/IRBs, trial participants, trial registries, journals, regulators)	___15__________
Consent or assent	26a	Who will obtain informed consent or assent from potential trial participants or authorised surrogates, and how (see Item 32)	8-9____________
	26b	Additional consent provisions for collection and use of participant data and biological specimens in ancillary studies, if applicable	___na__________
Confidentiality	27	How personal information about potential and enrolled participants will be collected, shared, and maintained in order to protect confidentiality before, during, and after the trial	_9___
Declaration of interests	28	Financial and other competing interests for principal investigators for the overall trial and each study site	__16___________
Access to data	29	Statement of who will have access to the final trial dataset, and disclosure of contractual agreements that limit such access for investigators	_____
Ancillary and post-trial care	30	Provisions, if any, for ancillary and post-trial care, and for compensation to those who suffer harm from trial participation	____________
Dissemination policy	31a	Plans for investigators and sponsor to communicate trial results to participants, healthcare professionals, the public, and other relevant groups (eg, via publication, reporting in results databases, or other data sharing arrangements), including any publication restrictions	_____15________
	31b	Authorship eligibility guidelines and any intended use of professional writers	_____na_________
	31c	Plans, if any, for granting public access to the full protocol, participant-level dataset, and statistical code	__16_________
**Appendices**			
Informed consent materials	32	Model consent form and other related documentation given to participants and authorised surrogates	_______
Biological specimens	33	Plans for collection, laboratory evaluation, and storage of biological specimens for genetic or molecular analysis in the current trial and for future use in ancillary studies, if applicable	__na___________

For peer review only - http://bmjopen.bmj.com/site/about/guidelines.xhtml

*It is strongly recommended that this checklist be read in conjunction with the SPIRIT 2013 Explanation & Elaboration for important clarification on the items. Amendments to the protocol should be tracked and dated. The SPIRIT checklist is copyrighted by the SPIRIT Group under the Creative Commons "Attribution-NonCommercial-NoDerivs 3.0 Unported" license.

For peer review only

For peer review only - http://bmjopen.bmj.com/site/about/guidelines.xhtml

BMJ Open

Protocol for a feasibility randomised trial of low intensity interventions for antenatal depression: ADAGIO trial comparing Interpersonal Counselling with Cognitive Behavioural Therapy.

Journal:	*BMJ Open*
Manuscript ID	bmjopen-2019-032649.R1
Article Type:	Protocol
Date Submitted by the Author:	05-Jul-2019

Complete List of Authors:	Ingram, Jenny; University of Bristol, Bristol Medical School, Centre for Academic Child Health<br>Johnson, Debbie; University of Bristol, Bristol Medical School, Centre for Academic Child Health<br>Johnson, Sarah; University Hospitals Bristol NHS Foundation Trust<br>O'Mahen, Heather; University of Exeter<br>Kessler, David; University of Bristol,<br>Taylor, Hazel; University Hospitals Bristol NHS Foundation Trust<br>Law, Roslyn; Anna Freud Centre<br>Round, Jeff; Institute of Health Economics<br>Ford, Jenny<br>Hopley, Rebecca; University of Exeter<br>Glynn, Joel; University of Bristol<br>Culpin, I; University of Bristol, Bristol Medical School<br>Evans, Jonathan; University Of Bristol, Bristol Medical School

**Primary Subject Heading**:	Mental health
Secondary Subject Heading:	Mental health
Keywords:	MENTAL HEALTH, Depression & mood disorders < PSYCHIATRY, Pregnancy

SCHOLARONE™
Manuscripts

Protocol for a feasibility randomised trial of low intensity interventions for antenatal depression: ADAGIO trial comparing Interpersonal Counselling with Cognitive Behavioural Therapy.

Jenny Ingram[1],

Debbie Johnson[1],

Sarah Johnson[2],

Heather O'Mahen[3],

David Kessler[4],

Hazel Taylor[5],

Roslyn Law[6],

Jeff Round[7],

Jenny Ford[2],

Becky Hopley[3],

Joel Glynn[8],

Iryna Culpin[4],

Jonathan Evans[4]

[1] Centre for Academic Child Health, Bristol Medical School, University of Bristol, UK.

[2] University Hospitals NHS Foundation Trust, Bristol, UK

[3] Department of Psychology, University of Exeter, UK

[4] Centre for Academic Mental Health, Bristol Medical School, University of Bristol, UK.

[5] Research Design Service- South West, University Hospitals NHS Foundation Trust, Bristol, UK

[6] Anna Freud National Centre for Children and Families, London, UK

[7] Institute of Health Economics, Alberta, Canada; and Health Economics Bristol, University of Bristol, UK

[8] Health Economics Bristol, Bristol Medical School, University of Bristol, UK

Corresponding author: Dr Jenny Ingram, Centre for Academic Child Health, University of Bristol, Bristol Medical School, 1-5 Whiteladies Road, Bristol BS8 1NU. 0117 428 3095;

Email: jenny.ingram@bristol.ac.uk.

For peer review only

For peer review only - http://bmjopen.bmj.com/site/about/guidelines.xhtml

ABSTRACT

Introduction. One in eight women suffer from depression during pregnancy. Currently low intensity brief treatment based on cognitive behavioural therapy (CBT) is the only talking treatment widely available in the NHS for mild and moderate depression. CBT involves identifying and changing unhelpful negative thoughts and behaviours to improve mood. Mothers in our patient advisory groups requested greater treatment choice. Interpersonal counselling (IPC)is a low intensity version of interpersonal therapy. It may have important advantages during pregnancy over CBT because it targets relationship problems, changes in role and previous losses (e.g. miscarriage). We aim to compare CBT and IPC for pregnant women with depression in a feasibility study.

Methods and analysis: A two-arm non-blinded randomised feasibility study of 60 women will be conducted in two UK localities. Women with depression will be identified through midwife clinics and ultrasound scanning appointments and randomised to receive six sessions of IPC or CBT. In every other way these women will receive usual care. Women thought to have severe depression will be referred for more intensive treatment. After 12 weeks we will measure women's mood, well-being, relationship satisfaction and use of health care. Women, their partners and staff providing treatments will be interviewed to understand whether IPC is an acceptable approach and whether changes should be introduced before applying to run a larger trial.
Several groups of patients with depression during pregnancy have contributed to our study design. A patient advisory group will meet and advise us during the study.

Ethics and dissemination: Study results will inform the design of a larger multicentre RCT. Our findings will be shared through public engagement events, papers and reports to organisations within the NHS. National Research Ethics Service Committee approved the study protocol.

Trial registration number: ISRCTN 11513120

For peer review only - http://bmjopen.bmj.com/site/about/guidelines.xhtml

Article summary.

Strengths and limitations of the study.

- This study uses a two-centre randomised controlled trial design to determine the feasibility of a definitive trial.
- Two methods of identifying participants will be used: through community midwives at booking and through ultrasound scanning clinics.
- Two talking therapies will be offered to assess the acceptability of these interventions during pregnancy.
- A process evaluation will explore the fidelity of intervention delivery and the experience of women, partners (or a significant other) and therapists.
- A definitive trial would be necessary to evaluate the effectiveness of the intervention.

Key words: Antenatal depression; Cognitive behavioural therapy; interpersonal counselling.

For peer review only

For peer review only - http://bmjopen.bmj.com/site/about/guidelines.xhtml

INTRODUCTION.

Antenatal depression is common, with a reported prevalence of 11%[1] and a point prevalence of up to 44% in certain populations.[2] Antenatal depression is associated with a range of poor outcomes including continuing depression into the postnatal period, reduced breastfeeding rates[3], infant developmental delay[4], and social/emotional problems, including depression in the offspring during adolescence.[5] Pregnancy does not appear to be protective against developing depression or relapsing in those with a previous history of depression.[6]

Currently, the Whooley questions are used to identify women who may have antenatal depression.[7] This screening method has high sensitivity (95%) and modest specificity (65%) for antenatal depression.[8] However, anecdotal evidence suggests that women are not always aware that they are referred for further help following a positive Whooley screen.[9]

There is limited evidence for the effectiveness of psychological interventions for antenatal depression[10], despite a widespread reluctance of mothers to take antidepressants during pregnancy[11] with 75% of women discontinuing antidepressant medication in the first trimester.[12] There is also concern amongst clinicians about prescribing antidepressant medication[13], largely due to a lack of evidence regarding their safety[14], indicating that psychological interventions are particularly important at this time.[10,15] One study found that women who discontinue antidepressant medication during pregnancy are five times more likely to experience a relapse in their depression requiring treatment.[6] The current treatment recommendation for mild to moderate depression, including depression that occurs during pregnancy, is a brief, one to one supported self-help approach using the principles of cognitive behavioural therapy (CBT) known as low intensity CBT.

Theoretical basis of CBT and limitations for this population.

The theoretical basis of CBT is that individuals prone to depression hold dysfunctional beliefs and therefore see the world through a negative filter.[16] Adversity, such as pregnancy, triggers these underlying beliefs and prone individuals then behave in a way that is consistent with these negative beliefs thus triggering and reinforcing depression. There is no specific relevance of this model to pregnancy and it relies on the skill of the therapist to adapt CBT to individual circumstances. Although there is a growing evidence base for ‘high intensity’ CBT delivered by experienced therapists for perinatal depression, ‘low intensity’ CBT is briefer and delivered by less experienced practitioners who have limited scope to adapt to the perinatal context and be flexible in its use. The use of high intensity CBT is limited within current clinical frameworks due to limitations in funding and therapist

For peer review only - http://bmjopen.bmj.com/site/about/guidelines.xhtml

availability.CBT has few explicit strategies to manage many of the problems that are common for women with antenatal depression, including role transitions and problems in relationships.

A recent review highlighted the need for more personalized therapies to treat perinatal depression, including interpersonal psychotherapy as a plausible treatment option.[17] Women in our patient advisory group consultations reported that CBT is 'too clinical' and 'inflexible' with practitioners not appearing to consider the circumstances of pregnancy. This may be one reason why the uptake of CBT treatment for antenatal depression is low. It has been reported that 14% of women with antenatal depression receive psychological treatment and only 5% with antenatal depression achieve remission following treatment.[18] Women who are pregnant receive lower rates of psychological intervention than those outside the perinatal period (30% vs 50%) despite an increased need for psychological treatment rather than drug treatment at this time.

Theoretical basis of Interpersonal Counselling (IPC) and advantages for treating antenatal depression.

IPC is a brief treatment which may be more appropriate for addressing the problems that depressed women have during pregnancy and postnatally. It is derived from interpersonal therapy, which holds that interpersonal relationships are a basic human need and attachment to key individuals provides a secure base from which to manage stressful events and conditions.[19] Problems in interpersonal relationships can trigger symptoms of depression, such as low mood or sleeplessness, and these symptoms further compromise relationships leading to a downward spiral.[20] This is particularly relevant to pregnancy as conflict in relationships and poor social support are the strongest risk factors for antenatal depression.[21]

IPC offers a more structured version of IPT that promotes understanding of depression through psychoeducation, problem solving and active involvement of the people in the person's life to provide support and promote recovery. IPC helps individuals develop useful strategies to manage interpersonal conflict and can involve the partner if appropriate. It also focuses on approaches that help manage changes in role, conflict, isolation and loss (i.e., miscarriage, still-birth, termination, previous loss of would be grandparents) and the impact of these on relationships. By directly approaching these issues, IPC addresses what is theoretically central to depression and what service users report are significant worries and concerns for them. The approach is simple and focused and could be provided by practitioners who have limited training or experience.

For peer review only - http://bmjopen.bmj.com/site/about/guidelines.xhtml

Currently there are limited data on the effectiveness of IPC.[14] It is critical to test its effectiveness because although almost half (43%, n=201,591 in 2015-16) of all individuals who receive a talking treatment in the NHS will receive a brief, “low-intensity” treatment, interpersonal brief treatment approaches are currently not available in the NHS.[22]

IPC has been clearly developed and rigorously manualised as an intervention allowing assessment of fidelity to the model. Although there have been very few studies of the effectiveness of IPC, and none in the UK, one study of depression in primary care in Italy found IPC to be more effective than antidepressants (SSRIs) particularly for those with less severe depression.[23] and a small UK pilot study found that IPC is an effective and acceptable treatment for young people with primarily depressive symptoms.[24] A small feasibility study of IPC for antenatal depression in the US amongst low income mothers indicated high satisfaction with IPC and some improvement in mood.[25]

There are therefore good reasons to hypothesise that IPC may be more acceptable at this time and particularly effective in treating antenatal depression. There is some evidence that interpersonal therapy (IPT), a high intensity therapy from which IPC is derived, is more acceptable than 'high intensity' CBT, with sessions more likely to be attended and that outcomes are better for IPT than 'high intensity' CBT in existing psychological treatment services.[22] We argue that evaluating this intervention for treating antenatal depression is particularly important because:
1) the model is particularly relevant to pregnancy and therefore the advantages of this approach over standard CBT treatment may be greater than at other times.[26]
2) psychological treatment provision is a priority at this time because of the potential risks of antidepressants during pregnancy and the costs of depression during pregnancy.[27]

Training low-intensity practitioners in IPC has the potential to offer women an acceptable, empirically valid option which may prove to be more effective than CBT and provide the NHS with a treatment option that may be both effective and cost-effective.

AIMS and OBJECTIVES.

Our overall aim is to improve psychological treatment for depression during pregnancy in order to improve outcomes for the mother, infant and the wider family. We aim to test whether six sessions of IPC are acceptable, effective and cost effective for treating mild to moderate antenatal depression compared to the most commonly provided existing

For peer review only - http://bmjopen.bmj.com/site/about/guidelines.xhtml

intervention, low intensity cognitive behavioural therapy (CBT). Both interventions are one to one therapies comprising up to six sessions.

The aim of this first study is to establish the feasibility and acceptability of conducting a full scale randomised controlled trial to compare the effectiveness of these two low intensity interventions for antenatal depression. Using a pragmatic study design operating within the existing care pathway, we will explore whether a full-scale trial using such a design is feasible. Study objectives are shown in Box 1.

Box 1: Detailed study objectives

To determine whether a full trial is feasible by assessing:

1) Whether staff who currently deliver low intensity interventions within routine psychological treatment services, known nationally as Improving Access to Psychological Treatment (IAPT) services, can reliably deliver this newly adapted therapy after brief, additional training.
2) Whether it is possible to recruit women from community midwife booking clinics and through screening at the ultrasound scanning clinics.
3) Whether it is feasible and acceptable to randomise women following assessment.
4) How acceptable IPC is to women, partners and those delivering the intervention relative to low intensity CBT, assessed through qualitative interviews.
5) Whether it is possible to collect sufficient outcome data, including those required to perform an economic evaluation.

METHODS AND ANALYSIS.

Study design.

This feasibility study over 21 months will be carried out in two centres (site A and site B), in preparation for a pragmatic fully powered RCT. Women with mild to moderate depression during pregnancy considered suitable for a low intensity intervention will be randomised 1:1 either to the usual low intensity treatment (CBT) or to a novel treatment called interpersonal counselling (IPC).

For peer review only - http://bmjopen.bmj.com/site/about/guidelines.xhtml

Study population, setting and recruitment plan

Women who are aged 18 years or over are eligible for inclusion between 10 and 24 weeks of pregnancy, with an Edinburgh Depression Scale (EPDS) score 10 or above and mild or moderate depression according to Clinical Interview Schedule Revised (CIS-R)[28], which gives an ICD-10 diagnosis, whether or not they are taking an antidepressant. Both primiparous and multiparous women will be eligible. Recruitment will continue for 9 months.

We will exclude women with psychotic illness, organic brain disorder, bipolar disorder, personality disorder, alcohol or substance dependency, which will be identified through self-report. Also excluded will be those judged to be at high suicide risk in assessor's judgement or from response to items on suicide in CIS-R or EPDS, those with severe depression according to CIS-R criteria, and those who have had CBT or IPT within the last 6 months. If women miscarry or have a termination during the trial, they will be offered the opportunity to continue with the treatment but will not be included in the rest of the study.

We will compare recruitment through two routes which have been used successfully in two previous trials of antenatal depression and antenatal anxiety:[29, 30]
Method 1: Women will be identified at community midwife booking clinics (around 8-10 weeks' gestation) through the routine screening undertaken for depression using the Whooley questions.[8] Those who answer 'yes' to either question and are considered by the midwife to be appropriate for further assessment for talking therapy, will be asked for their consent to be contacted by the research team about the study.

Method 2: Women will also be identified from ultrasound scan clinics where they will be given study information, eligibility screening questions (EPDS) and a form to indicate their willingness to be contacted by the research team for assessment if screening positive (EPDS 10 or above). This approach will provide a good opportunity to recruit disadvantaged groups, as attendance at scanning clinics does not appear to be socially patterned.[31] Where needed, interpreters will be used to ensure that those who do not have English as a first language can be offered therapy.

Figure 1 shows the flow of participants through the trial.

Assessment and randomisation.

A researcher will conduct a telephone assessment with women who consent to be contacted. At a subsequent face to face meeting the researcher will establish eligibility for

For peer review only - http://bmjopen.bmj.com/site/about/guidelines.xhtml

the trial, obtain written consent and collect baseline data. Partners will also be asked for their consent either in person at the baseline visit, online or by post.

Randomisation will be carried out remotely by Bristol Randomised Trials Collaboration randomisation service. It will be stratified by recruiting centre and minimised by parity (with random block sizes).

Treatment arms

We have embedded the treatments within existing psychological treatment services available in primary care (Improving Access to Psychological Therapies: IAPT). For women with mild or moderate depression during pregnancy considered suitable for a low intensity intervention, we will compare up to six sessions of either low intensity CBT or IPC.

Training and supervision for practitioners

To avoid potential selection bias, 12 psychological wellbeing practitioners will be randomised, six to be trained in IPC and six to continue delivering low intensity CBT.

IAPT low intensity (brief therapy) practitioners who agreed to take part in the study and are randomised to the IPC group will receive three days of training from RL (co-applicant; chair of Interpersonal Therapy (IPT) UK; experienced, certified IPT/IPC trainer). Supervisors for the practitioners, who are already trained in IPT (the model from which IPC is derived) will attend the training days. The practitioners will be required to audio record cases, which will be assessed by their supervisor to ensure competence and fidelity to the model. Supervision will be provided weekly initially, and then fortnightly once practitioners are more familiar with IPC. Those delivering the six sessions of CBT will be given refresher training and have fortnightly case supervision as is usual practice for CBT. Supervisors will rate adherence from a checklist and feedback to trainees. These ratings will be used to assess fidelity.

Data Collection and management

All data collected and analysed during the study will be pseudo-anonymised using a unique identifier. A record of trial participants' names and contact details and assigned trial numbers will be maintained by the trial coordinator and stored separately and securely for administrative purposes. Study data collected by the research team will be recorded on study specific data collection forms (CRFs). Data will then be entered onto a REDCap database.

For peer review only - http://bmjopen.bmj.com/site/about/guidelines.xhtml

Baseline Measurements

Baseline data will be collected at an initial face-to-face assessment with women and will include measures of mood, quality of life, quality of relationship with partner and antenatal attachment. Partners will also be invited to complete a depression rating scale.

Follow-up Measurements

Assessments will be completed either online by participants or over the telephone with a researcher 12 weeks following randomisation. This allows time for women to be allocated to therapy and to complete the sessions. Non-responders will receive two automatic online or telephone reminders one week apart, with attempts to collect data by telephone a week later if necessary.

Measures at baseline and 12-weeks

1) CIS-R a computerised structured psychiatric interview (at baseline only)
2) Edinburgh Postnatal Depression Scale (EPDS)[32] continuous and binary scores from women and their partners. The scale, sometimes known as the Edinburgh Depression Scale, was developed for postnatal depression but is widely used during pregnancy and has been validated outside the postnatal period and for men.[33, 34]
3) The Revised Dyadic Adjustment Scale[35] assesses partner satisfaction.
4) Maternal Antenatal Attachment Scale.[36]
5) Health economic measures outlined below (EQ-5D-5L, ReQol10).
6) At 12-weeks only: The number of sessions attended, number that include the partner, whether step up to more intense psychological intervention is needed, use of medication, and use of secondary mental health services.

Blinding

It will not be possible for assessors or participants to be blind to allocation however the statistician will be blind to allocation of participants.

Outcomes

The primary outcome will be the proportion of eligible women successfully recruited to the point of randomisation.

We will assess numbers and proportions of participants:

- Recruited for assessment (comparing two methods of recruitment),
- Randomised
- Completing the course of treatment

For peer review only - http://bmjopen.bmj.com/site/about/guidelines.xhtml

• Completing follow up measures

• Requiring 'step up' to a higher intensity intervention.

We will assess the acceptability of the recruitment method, intervention and study design through a series of in-depth interviews with participants and IAPT practitioners.

The primary outcome for a future trial is likely to be change in EPDS score but we will also consider the other secondary outcomes collected in this trial.

Sample size determination.

As this is a feasibility study, the sample size should be sufficient to measure feasibility parameters and data completeness with adequate precision.

There are around 3000 pregnancies per year at site A and 1500 at site B (total 4500) in the relevant IAPT catchment areas, giving a total of approximately 3375 pregnancies in the nine-month recruitment period. Assuming 10% of these have mild-moderate antenatal depression (338) and recruiting through both midwives booking appointments and ultrasound scanning clinics we aim to include 60 women. This target of 60 subjects from 338 potentially eligible women (17.8%) gives a 95% confidence interval for recruitment between 13.9% and 22.3%

Economic evaluation

A full economic evaluation is not possible based on the results of this feasibility study. Within the feasibility study we assess whether and how necessary data can be collected. We will pilot methods for collecting resource use data in this population and use the results to plan the future main trial. Intervention delivery resource will be recorded.

The main economic outcome measure collected will be the EQ-5D-5L, a generic preference-based measure of health.[37] Recognising that non-health dimensions of wellbeing were important to our PPI group, we will also collect data on the ReQoL10 instrument, an alternative preference-based outcome tool that includes domains beyond health and has been developed specifically for use in groups with mental health problems (www.requol.org.uk). Resource use data will be collected online or by telephone as part of the 12-week follow-up. We will ask participants to report resource use during the time enrolled on the study.

For peer review only - http://bmjopen.bmj.com/site/about/guidelines.xhtml

Qualitative study.

We will collect qualitative data through interviews to assist in determining the feasibility and acceptability of the intervention and trial design. We will explore the acceptability of the intervention to women receiving IPC and CBT; the feasibility of recruitment and follow up. We will conduct semi-structured interviews either face to face or on the phone. It is anticipated interviews will last between 30 minutes and 1 hour. Topic guides will be informed by the research literature, team discussions and input from PPI. We will interview the following groups:

A) *Women in the treatment arms*.

These will be conducted at the completion of either IPC (10-12 women) or CBT (5-6 women). Purposive maximum variation sampling will ensure that women from different age groups, socioeconomic status, ethnicity and different levels of engagement with the intervention are selected from both study sites. Interviews will focus on the acceptability and perceived effectiveness of the talking therapy and explore views on the recruitment process; helpful and challenging aspects of the intervention; and the appropriateness of the outcome measures being used.

B) *Partners (or significant others) of those receiving IPC or CBT.*

Interviews will be conducted with 5 or 6 partners focusing on the acceptability and perceived effectiveness of the intervention and explore ways they feel their partners have benefited from the talking therapy.

C) *Participants who drop out.*

We will seek the views of participants who withdrew or did not attend the intervention to understand their reasons for dropping out and whether continuing participation (and engagement in the intervention) could be supported. These will be short telephone interviews and we would attempt to contact them up to three times.

D) *Staff interviews.*

Interviews (6-8) with practitioners in the IPC arm, their supervisors and community midwives, will be carried out at the end of the intervention and focus on the acceptability, strengths and weaknesses of the intervention.

For peer review only

Data Analyses

Quantitative Data analysis

As this is a feasibility trial no formal statistical testing will be carried out. Instead the analysis will focus on reporting data that will be used for planning and for assessing the feasibility of the full trial.

A CONSORT flow diagram will be produced. Proportions with 95% confidence intervals calculated using the Exact Binomial Method will be produced for:

- Participants consented.
- Participants who are randomised with completed baseline measures.
- Participants randomised to IPC who complete it.
- Participants randomised to low intensity CBT who complete it.
- Randomised participants lost to follow-up.
- Randomised participants who require 'step up' to a higher intensity intervention.
- Randomised participants who have complete outcome data.

Baseline characteristics and demographic characteristics will be tabulated by treatment group (defined by intention to treat) and overall. Means or medians together with appropriate measures of dispersion will be reported for continuous measures and proportions for binary measures. The follow-up outcome data (namely the EPDS, Revised Dyadic Adjustment Scale, Maternal Antenatal Attachment Scale, number of sessions attended, number of sessions partner attended, medication use and secondary health service use) will be reported in the same way. Plots will be used to examine the distribution of continuous outcomes.

Qualitative data analysis

All interviews will be audio-recorded, transcribed verbatim and anonymised. Thematic analysis methods[38] will be used with NVivo to aid data management. Interview transcripts will be read and re-read individually, from which an initial coding framework will be developed. Team members will meet to discuss the preliminary coding framework and themes to ensure that the emerging analysis is trustworthy and credible. This framework will be added to and refined, with coded material regrouped as new data from subsequent interviews are gathered.

For peer review only - http://bmjopen.bmj.com/site/about/guidelines.xhtml

Patient and public involvement / Patient Advisory Group (PAG)

Discussions with women who have perinatal mental health problems and are currently using services, colleagues running voluntary sector perinatal mental health services and feedback from public engagement events, have all highlighted the need to improve psychological treatment services available to women and their partners during pregnancy and following childbirth. We asked women attending an antenatal group for those with mental health problems in pregnancy what they thought would have helped them most and they highlighted the need for a therapy that is more specific to pregnant women. Some reported that current treatment offered (CBT) was 'too clinical' and those providing the treatment made little reference to pregnancy or worries about coping with a young baby. We have two collaborators who run voluntary sector organisations providing help to women with mental health problems during the perinatal period. One has been involved in the development of the ideas surrounding this and the other has been providing advice about women's experience of local psychological treatment services, as well as commenting on the proposal and several aspects of the design. At two public engagement events held in Bristol we discussed services for families and how these could be improved. At one event for fathers whose partners struggled with their mood and anxiety during pregnancy or following child birth they gave a clear message that they felt excluded and even treated with suspicion by services. Fathers welcomed any psychological treatment which might include them and focus on improving the relationship with their partner. Six women who have been attending either an antenatal group aimed at promoting emotional wellbeing or postnatal drop-in sessions run by the voluntary sector, have agreed to form a Patient Advisory Group (PAG) meeting three times during the study.

The PAG members will assist in the development of patient facing materials, advise on recruitment issues, inform the development of the topic guide for the qualitative interviews and discuss and help interpret the results including the decision on whether to proceed to a full trial. They will be offered a study specific induction pack which will include the INVOLVE materials and relevant study information. Training workshops run by People and Research West of England will also be offered to them. PAG members will have their travel expenses and meeting time reimbursed with vouchers. Our findings will be presented in lay terms at a PAG meeting and they will advise us on routes for dissemination to patient groups.

For peer review only

ETHICS, MONITORING AND DISSEMINATION

This manuscript is based on Protocol V.3.0 dated 14/06/2019. The study received North of Scotland Research Ethics Committee (REC) approval on October 29th 2018 and Health Research Authority approval on November 14th 2018. The trial will be conducted in accordance with the protocol, the principles of the Declaration of Helsinki and ICH GCP. Any amendments of the protocol will be submitted to the REC for approval. On request, the study investigators and their institutions will permit trial-related monitoring and audits by the Sponsor and relevant Research Ethics Committee by providing direct access to source data and other documents (i.e. patients' hospital notes). The University of Bristol holds the relevant insurance for this study and is the nominated sponsor for this study.

A Trial Steering Committee (TSC) has been convened to provide overall supervision of the trial and ensure it is in accordance with the principles of good clinical practice and relevant regulations. The TSC agreed the trial protocol and will agree any protocol amendments. The TSC also provide advice to the investigators on all aspects of the trial including aspects of safety and monitoring of serious adverse events. The TSC is chaired by Prof Paul Ramchandani with three independent members who have expertise in clinical psychology and perinatal mental health, midwifery for NHS England, and statistics.

Dissemination.

A lay summary of the study is available on the NIHR website. Results of this feasibility study will be publicly available through open access publication in a peer-reviewed journals and presented at relevant conferences and research meetings. The PPI groups will contribute to the dissemination plan and assist in the production of the lay summaries.

Word count 3979

Acknowledgements The authors would like to thank the lay representatives who contribute to the trial management and steering groups and the women who contributed to the intervention design.

Author Contributions: JE is the chief investigator and JI drafted the paper with critical input from all authors. All authors contributed to study design and development. JE, JI, HO'M, DK,

For peer review only - http://bmjopen.bmj.com/site/about/guidelines.xhtml

HT, JR, RL, IC and JF contributed to the inception of the study. DJ, BH, SJ and JG contributed to revisions of the protocol. DJ, BH are currently recruiting participants to the trial. All authors read, commented on and approved the final manuscript.

Funding: This work is supported by the National Institute for Health Research (NIHR) Research for Patient Benefit research programme grant number PB-PG-1216-20027.

Disclaimer: The views expressed are those of the authors and not necessarily those of the NHS, the NIHR or the Department of Health.

Competing interests: None

Provenance and peer review: Not commissioned; externally peer reviewed.

Data sharing statement: The results of this feasibility study will inform the planning of a definitive randomised controlled trial, including whether it can be conducted, by assessing rates of recruitment, retention and data collection. Data will be made available upon reasonable request.

Trial status: Ongoing data collection.

Trial sponsor: University of Bristol. Bristol BS1 5DD: research-governance@bristol.ac.uk

REFERENCES.

1. Howard, L.M., Ryan, E.G., Trevillion, K., Anderson, F., Bick, D., Bye, A. et al. (2018). Accuracy of the Whooley questions and the Edinburgh Postnatal Depression Scale in identifying depression and other mental disorders in early pregnancy. British Journal of Psychiatry, 212: 50-56.

2. Gavin, N. I., Gaynes, B. N., Lohr, K. N., Meltzer-Brody, S., Gartlehner, G., & Swinson, T. (2005). Perinatal depression: a systematic review of prevalence and incidence. *Obstetrics & Gynecology*, *106*(5, Part 1), 1071-1083.

3. Grigoriadis, S., Vonderporten, E.H., Mamisashvili, L., Tomlinson, G., Dennis, C.L., Koren, G. et al. (2013). The impact of maternal depression during pregnancy on perinatal outcomes: A systematic review and meta-analysis. Journal of Clinical Psychiatry, 74(4): 321

4. Pedersen, L.H., Henriksen, T.B. and Olsen, J. (2010) Fetal exposure to antidepressants and normal milestone development at 6 and 19 months of age. Pediatrics, 125(3): 600-608

For peer review only - http://bmjopen.bmj.com/site/about/guidelines.xhtml

5. Madigan, S., Oatley, H., Racine, N., Fearon, R.M.P., Schumacher, L., Akbari, E. et al. (2018). A meta-analysis of maternal prenatal depression and anxiety on child socioemotional development. Journal of the American Academy of Child and Adolescent Psychiatry, 57(9): 645

6. Cohen, L.S., Altshuler, L.L., Harlow, B.L., Nonacs, R., Newport, D.J., Viguera, A.C. et al. (2006). Relapse of major depression during pregnancy in women who maintain or discontinue antidepressant treatment. Journal of the American Medical Association, 295(5): 499-507.

7. NICE Clinical guideline 192 (2014) Antenatal and postnatal mental health: clinical management and service guidance.

8. Bosanquet, K., Bailey, D., Gilbody, S., Harden, M., Manea, H., Nutbrown, S. and McMillan, D. (2015). Diagnostic accuracy of the Whooley questions for the identification of depression: A diagnostic meta-analysis. *British Medical Journal Open.* 5(12)

9. Darwin, Z., McGowan, L. and Edozien, L.C. (2015). Antenatal mental health referrals: Review of local clinical practice and pregnant women's experiences in England. Midwifery, 31(3): 17-22

10. Dennis, C. L., Ross, L. E., & Grigoriadis, S. (2007). Psychosocial and psychological interventions for treating antenatal depression. Cochrane Database of Systematic Reviews 2007, Issue 3. Art. No.: CD006309. DOI: 10.1002/14651858.CD006309.pub2.

11. Arch, J.J. (2014). Cognitive behavioral therapy and pharmacotherapy for anxiety: Treatment preferences and credibility among pregnant and non-pregnant women. *Behaviour Research and Therapy* 52:53-60

12. Hayes, R.M., Wu, P.S., Shelton, R.C., Cooper, W.O., Dupont, W.D., Mitchel, E. and Hartert, T.V. (2012) Maternal antidepressant use and adverse outcomes: A cohort study of 228876 pregnancies. American Journal of Obstetrics and Gynecology, 207(1)

13. Bilszta, J.L.C., Tsuchiya, S., Han, K., Buist, A.E., Einarson, A. (2011). Primary care physicians' attitudes and practices regarding antidepressant use during pregnancy: A survey of two countries. *Archives of Womens Mental Health.* 14(1): 71-75

14. Chisolm, M.S. and Payne, J.L. (2016) Management of psychotropic drugs during pregnancy. British Medical Journal, 352:h5918

15. Sockol, L. E., Epperson, C. N., & Barber, J. P. (2011). A meta-analysis of treatments for perinatal depression. Clinical Psychology Review, 31(5), 839-849.

16. Beck, A.T. (1967) Depression: Clinical, Experimental, and Theoretical Aspects. New York: Harper & Row.

17. Johansen, S.L., Robakis, T.K., Williams, K.E. and Rasgon, N.L. (2019) Management of perinatal depression with non-drug interventions. British Medical Journal, 364:l322.

18. Cox, E.Q., Sowa, N.A., Meltzer-Brody, S.E. and Gaynes, B.N. (2016) The perinatal depression treatment cascade: Baby steps toward improving outcomes. Journal of Clinical Psychiatry, 77(9):1189-1200.

For peer review only

For peer review only - http://bmjopen.bmj.com/site/about/guidelines.xhtml

19. Ravitz, P., Watson, P., Lawson, A., Contatino, M.J., Bernecker, S., Park, J. and Swartz, H.A. (2019) Interpersonal psychotherapy: A scoping review and historical perspective (1974-2017). Harvard Review of Psychiatry. Accessed online via PMID 30883446.

20. Lipsitz, J. D., & Markowitz, J. C. (2013). Mechanisms of change in interpersonal therapy (IPT). Clinical psychology review, 33(8), 1134-1147.

21. Lancaster, C. A., Gold, K. J., Flynn, H. A., Yoo, H., Marcus, S. M., & Davis, M. M. (2010). Risk factors for depressive symptoms during pregnancy: a systematic review. American Journal of Obstetrics and Gynecology, 202(1), 5-14.

22. NHS Digital 2016: Psychological Therapies: Annual Report on the use of IAPT services, England, 2015-16

23. Menchetti, M., Rucci, P., Bortolotti, B., Bombi, A., Scocco, P., Kraemer, H. C.,Berardi D, DEPICS group. (2013). Moderators of remission with interpersonal counselling or drug treatment in primary care patients with depression: randomised controlled trial. The British Journal of Psychiatry. 204(2): 144-150.

24. Wilkinson PO, Cestaro V, Pinchen I. Pilot mixed-methods evaluation of interpersonal counselling for young people with depressive symptoms in non-specialist services *Evidence-Based Mental Health* 2018;**21:**134-138.

25. Lenze, Shannon N., and Mary Anne Potts. (2017) Brief Interpersonal Psychotherapy for depression during pregnancy in a low-income population: A randomized controlled trial Journal of Affective Disorders 210: 151-157.

26. Stuart, S. (2012). Interpersonal psychotherapy for postpartum depression. Clinical Psychology & Psychotherapy, 19(2), 134-140.

27. Bauer, A., Parsonage, M., Knapp, M., Lemmi, V., & Adelaja, B. (2014). Costs of perinatal mental health problems. London School of Economics and Political Science.

28. Lewis G, Pelosi AJ, Araya R, Dunn G: (1992) Measuring psychiatric-disorder in the community – a standardized assessment for Use by Lay interviewers. Psychol Med. 22 (2): 465-486.

29. Burns, A., O'Mahen, H., Baxter, H., Bennert, K., Wiles, N., Ramchandani, P., ... & Evans, J. (2013). A pilot randomised controlled trial of cognitive behavioural therapy for antenatal depression.BMC Psychiatry, 13(1), 33.

30. Wilkinson, E. L., O'Mahen, H. A., Fearon, P., Halligan, S., King, D. X., Greenfield, G., ... & Ramchandani, P. G. (2016). Adapting and testing a brief intervention to reduce maternal anxiety during pregnancy (ACORN): study protocol for a randomised controlled trial. Trials, 17(1), 156.

31. Redshaw, M., & Henderson, J. (2013). Fathers' engagement in pregnancy and childbirth: evidence from a national survey. BMC Pregnancy and Childbirth, 13(1), 70.

32. Cox, J. L., Holden, J. M., & Sagovsky, R. (1987). Detection of postnatal depression. Development of the 10-item Edinburgh Postnatal Depression Scale. The British Journal of Psychiatry, 150(6), 782-786.

33. Hewitt C, Gilbody S, Brealey S, et al. (2009) Methods to identify postnatal depression in primary care: an integrated evidence synthesis and value of information analysis. Health

For peer review only - http://bmjopen.bmj.com/site/about/guidelines.xhtml

Technol Assess.13(36):1-145, 147-230.

34. Edmondson OJ, Psychogiou L, Vlachos H, Netsi E, Ramchandani PG. (2010) Depression in fathers in the postnatal period: assessment of the Edinburgh Postnatal Depression Scale as a screening measure. J Affect Disord.125(1-3):365-368.

35. Busby DM, Christensen C, Crane DR, Larson JH. (1995). A revision of the dyadic adjustment scale for use with distressed and nondistressed couples: construct hierarchy and multidimensional scales. Journal of Marital and Family Therapy; 21(3):289-308.

36. Condon JT. (1993) The assessment of antenatal emotional attachment: development of a questionnaire instrument. Br J Med Psychol; 66 (Pt 2): 167-183

37. Herdman, M., Gudex, C., Lloyd, A., Janssen, M., Kind, P., Parkin, D., Bonsel, G., Badia, X. (2011) Development and preliminary testing of the new five-level version of EQ-5D (EQ-5D-5L) *Quality of Life Research,* 20(10), pp1727-1736.

38. Braun, V., & Clarke, V. (2006), Using thematic analysis in psychology. *Qualitative Research in Psychology*, *3*, 77-101.

Figure 1. ADAGIO study flow diagram

For peer review only - http://bmjopen.bmj.com/site/about/guidelines.xhtml

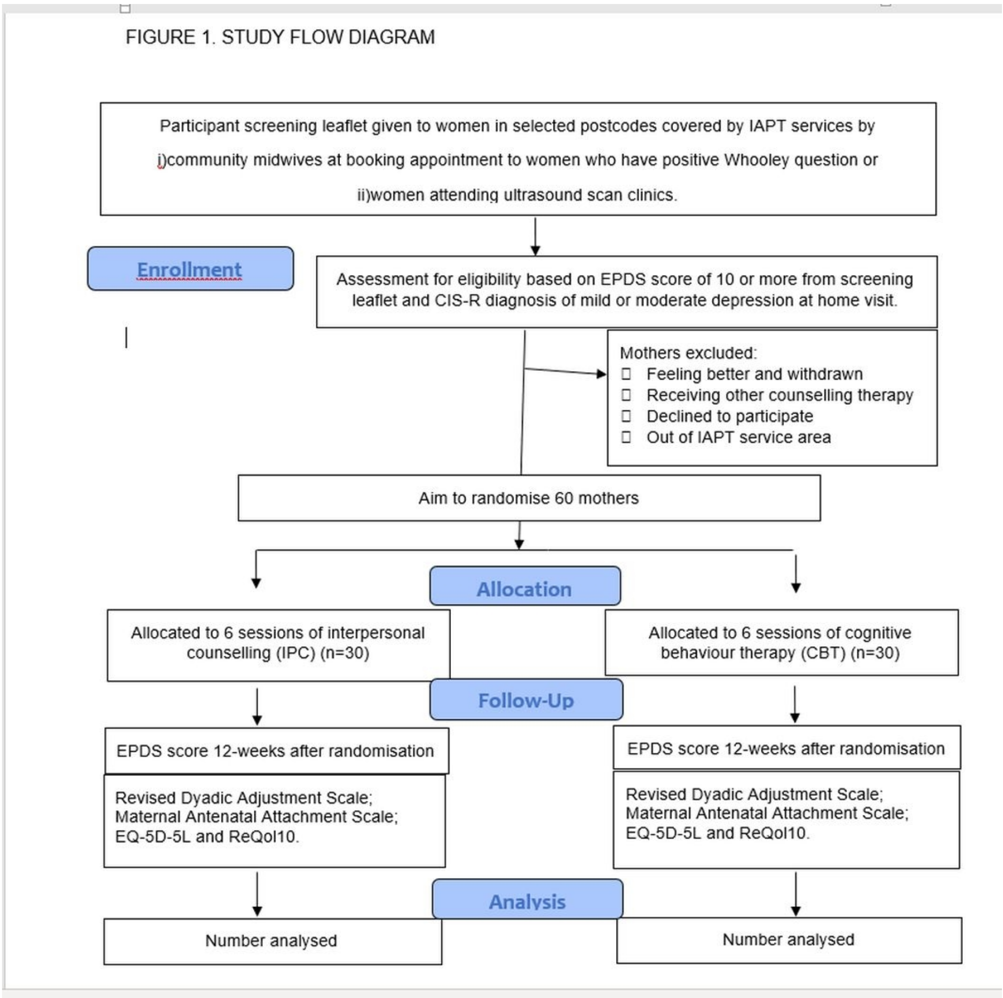

Figure 1 ADAGIO study flow diagram

90x89mm (300 x 300 DPI)

SPIRIT

STANDARD PROTOCOL ITEMS: RECOMMENDATIONS FOR INTERVENTIONAL TRIALS

SPIRIT 2013 Checklist: Recommended items to address in a clinical trial protocol and related documents*

Section/item	Item No	Description	Addressed on page number
**Administrative information**			
Title	1	Descriptive title identifying the study design, population, interventions, and, if applicable, trial acronym	___1_________
Trial registration	2a	Trial identifier and registry name. If not yet registered, name of intended registry	__2___________
	2b	All items from the World Health Organization Trial Registration Data Set	____N/A________ _
Protocol version	3	Date and version identifier	___15__________
Funding	4	Sources and types of financial, material, and other support	__16___________
Roles and responsibilities	5a	Names, affiliations, and roles of protocol contributors	___15__________
	5b	Name and contact information for the trial sponsor	___16__________
	5c	Role of study sponsor and funders, if any, in study design; collection, management, analysis, and interpretation of data; writing of the report; and the decision to submit the report for publication, including whether they will have ultimate authority over any of these activities	____15/16__
	5d	Composition, roles, and responsibilities of the coordinating centre, steering committee, endpoint adjudication committee, data management team, and other individuals or groups overseeing the trial, if applicable (see Item 21a for data monitoring committee)	__15________

For peer review only

**Introduction**			
Background and rationale	6a	Description of research question and justification for undertaking the trial, including summary of relevant studies (published and unpublished) examining benefits and harms for each intervention	_4-6_________
	6b	Explanation for choice of comparators	__6___________
Objectives	7	Specific objectives or hypotheses	6-7_________
Trial design	8	Description of trial design including type of trial (eg, parallel group, crossover, factorial, single group), allocation ratio, and framework (eg, superiority, equivalence, noninferiority, exploratory)	_7 (in Methods)_
**Methods: Participants, interventions, and outcomes**			
Study setting	9	Description of study settings (eg, community clinic, academic hospital) and list of countries where data will be collected. Reference to where list of study sites can be obtained	_7________
Eligibility criteria	10	Inclusion and exclusion criteria for participants. If applicable, eligibility criteria for study centres and individuals who will perform the interventions (eg, surgeons, psychotherapists)	_8
Interventions	11a	Interventions for each group with sufficient detail to allow replication, including how and when they will be administered	7, 9__
	11b	Criteria for discontinuing or modifying allocated interventions for a given trial participant (eg, drug dose change in response to harms, participant request, or improving/worsening disease)	_8____________
	11c	Strategies to improve adherence to intervention protocols, and any procedures for monitoring adherence (eg, drug tablet return, laboratory tests)	_9_______
	11d	Relevant concomitant care and interventions that are permitted or prohibited during the trial	_n/a___________ _
Outcomes	12	Primary, secondary, and other outcomes, including the specific measurement variable (eg, systolic blood pressure), analysis metric (eg, change from baseline, final value, time to event), method of aggregation (eg, median, proportion), and time point for each outcome. Explanation of the clinical relevance of chosen efficacy and harm outcomes is strongly recommended	__10-11_______

For peer review only - http://bmjopen.bmj.com/site/about/guidelines.xhtml

Participant timeline	13	Time schedule of enrolment, interventions (including any run-ins and washouts), assessments, and visits for participants. A schematic diagram is highly recommended (see Figure)	_8-11_________
Sample size	14	Estimated number of participants needed to achieve study objectives and how it was determined, including clinical and statistical assumptions supporting any sample size calculations	_11__________
Recruitment	15	Strategies for achieving adequate participant enrolment to reach target sample size	__8___________
**Methods: Assignment of interventions (for controlled trials)**			
Allocation:			
Sequence generation	16a	Method of generating the allocation sequence (eg, computer-generated random numbers), and list of any factors for stratification. To reduce predictability of a random sequence, details of any planned restriction (eg, blocking) should be provided in a separate document that is unavailable to those who enrol participants or assign interventions	__9___________
Allocation concealment mechanism	16b	Mechanism of implementing the allocation sequence (eg, central telephone; sequentially numbered, opaque, sealed envelopes), describing any steps to conceal the sequence until interventions are assigned	__9___________
Implementation	16c	Who will generate the allocation sequence, who will enrol participants, and who will assign participants to interventions	___8-9_______
Blinding (masking)	17a	Who will be blinded after assignment to interventions (eg, trial participants, care providers, outcome assessors, data analysts), and how	_10__
	17b	If blinded, circumstances under which unblinding is permissible, and procedure for revealing a participant's allocated intervention during the trial	___na__________
**Methods: Data collection, management, and analysis**			
Data collection methods	18a	Plans for assessment and collection of outcome, baseline, and other trial data, including any related processes to promote data quality (eg, duplicate measurements, training of assessors) and a description of study instruments (eg, questionnaires, laboratory tests) along with their reliability and validity, if known. Reference to where data collection forms can be found, if not in the protocol	_10-11__

	18b	Plans to promote participant retention and complete follow-up, including list of any outcome data to be collected for participants who discontinue or deviate from intervention protocols	__10___________
Data management	19	Plans for data entry, coding, security, and storage, including any related processes to promote data quality (eg, double data entry; range checks for data values). Reference to where details of data management procedures can be found, if not in the protocol	___9__________
Statistical methods	20a	Statistical methods for analysing primary and secondary outcomes. Reference to where other details of the statistical analysis plan can be found, if not in the protocol	_13_______
	20b	Methods for any additional analyses (eg, subgroup and adjusted analyses)	_na__________
	20c	Definition of analysis population relating to protocol non-adherence (eg, as randomised analysis), and any statistical methods to handle missing data (eg, multiple imputation)	__na___________
**Methods: Monitoring**			
Data monitoring	21a	Composition of data monitoring committee (DMC); summary of its role and reporting structure; statement of whether it is independent from the sponsor and competing interests; and reference to where further details about its charter can be found, if not in the protocol. Alternatively, an explanation of why a DMC is not needed	_15______
	21b	Description of any interim analyses and stopping guidelines, including who will have access to these interim results and make the final decision to terminate the trial	_na____________
Harms	22	Plans for collecting, assessing, reporting, and managing solicited and spontaneously reported adverse events and other unintended effects of trial interventions or trial conduct	__15__________
Auditing	23	Frequency and procedures for auditing trial conduct, if any, and whether the process will be independent from investigators and the sponsor	___15__________
**Ethics and dissemination**			
Research ethics approval	24	Plans for seeking research ethics committee/institutional review board (REC/IRB) approval	_15____________

For peer review only - http://bmjopen.bmj.com/site/about/guidelines.xhtml

Protocol amendments	25	Plans for communicating important protocol modifications (eg, changes to eligibility criteria, outcomes, analyses) to relevant parties (eg, investigators, REC/IRBs, trial participants, trial registries, journals, regulators)	___15__________
Consent or assent	26a	Who will obtain informed consent or assent from potential trial participants or authorised surrogates, and how (see Item 32)	8-9____________
	26b	Additional consent provisions for collection and use of participant data and biological specimens in ancillary studies, if applicable	___na__________
Confidentiality	27	How personal information about potential and enrolled participants will be collected, shared, and maintained in order to protect confidentiality before, during, and after the trial	_9___
Declaration of interests	28	Financial and other competing interests for principal investigators for the overall trial and each study site	__16___________
Access to data	29	Statement of who will have access to the final trial dataset, and disclosure of contractual agreements that limit such access for investigators	___15__
Ancillary and post-trial care	30	Provisions, if any, for ancillary and post-trial care, and for compensation to those who suffer harm from trial participation	___na_________
Dissemination policy	31a	Plans for investigators and sponsor to communicate trial results to participants, healthcare professionals, the public, and other relevant groups (eg, via publication, reporting in results databases, or other data sharing arrangements), including any publication restrictions	_____15________
	31b	Authorship eligibility guidelines and any intended use of professional writers	_____na_________
	31c	Plans, if any, for granting public access to the full protocol, participant-level dataset, and statistical code	__16_________
**Appendices**			
Informed consent materials	32	Model consent form and other related documentation given to participants and authorised surrogates	___attached____
Biological specimens	33	Plans for collection, laboratory evaluation, and storage of biological specimens for genetic or molecular analysis in the current trial and for future use in ancillary studies, if applicable	__na___________

For peer review only - http://bmjopen.bmj.com/site/about/guidelines.xhtml

*It is strongly recommended that this checklist be read in conjunction with the SPIRIT 2013 Explanation & Elaboration for important clarification on the items. Amendments to the protocol should be tracked and dated. The SPIRIT checklist is copyrighted by the SPIRIT Group under the Creative Commons "Attribution-NonCommercial-NoDerivs 3.0 Unported" license.

For peer review only
