# Supplementary material for: Protocol for a feasibility randomised trial of low-intensity interventions for antenatal depression: ADAGIO trial comparing interpersonal counselling with cognitive behavioural therapy
Source: BMJ Open. 2019 Aug 18;9(8):e032649. doi: 10.1136/bmjopen-2019-032649 (PMC6701625; doi:10.1136/bmjopen-2019-032649)
Supplement: Reviewer comments [file bmjopen-2019-032649.reviewer_comments.pdf]

PEER REVIEW HISTORY

BMJ Open publishes all reviews undertaken for accepted manuscripts. Reviewers are asked to complete a checklist review form (**http://bmjopen.bmj.com/site/about/resources/checklist.pdf**) and are provided with free text boxes to elaborate on their assessment. These free text comments are reproduced below.

ARTICLE DETAILS

**TITLE (PROVISIONAL)**	Protocol for a feasibility randomised trial of low intensity interventions for antenatal depression: ADAGIO trial comparing Interpersonal Counselling with Cognitive Behavioural Therapy.
**AUTHORS**	Ingram, Jenny; Johnson, Debbie; Johnson, Sarah; O'Mahen, Heather; Kessler, David; Taylor, Hazel; Law, Roslyn; Round, Jeff; Ford, Jenny; Hopley, Rebecca; Glynn, Joel; Culpin, I; Evans, Jonathan

This article was not externally reviewed at BMJ Open. Protocols that have been independently assessed prior to submission to BMJ Open are usually fast-tracked to publication on the grounds that further substantial changes will not be possible. This independent assessment will usually be external, independent review for both a substantial grant award from a non-commercial or government funder and ethics approval.
